# Response to anti-IL17 therapy in inflammatory disease is not strongly impacted by genetic background

**DOI:** 10.1016/j.ajhg.2023.08.010

**Published:** 2023-09-01

**Authors:** Cong Zhang, Konstantin Shestopaloff, Benjamin Hollis, Chun Hei Kwok, Claudia Hon, Nicole Hartmann, Chengeng Tian, Magdalena Wozniak, Luis Santos, Dominique West, Stephen Gardiner, Ann-Marie Mallon, Aimee Readie, Ruvie Martin, Thomas Nichols, Michael T. Beste, Jonas Zierer, Enrico Ferrero, Marc Vandemeulebroecke, Luke Jostins-Dean

**Affiliations:** 1 China Novartis Institutes for Bio-medical Research CO., Shanghai, China; 2 Big Data Institute, Li Ka Shing Centre for Health Information and Discovery, Nuffield Department of Medicine, University of Oxford, Oxford, UK; 3 Department of Statistics, University of Oxford, Oxford, UK; 4 Kennedy Institute of Rheumatology, University of Oxford, Oxford, UK; 5 Nuffield Department of Population Health, University of Oxford, Oxford, UK; 6 Novartis Institutes for BioMedical Research, 220 Massachusetts Avenue, Cambridge, MA 02139, USA; 7 Novartis Pharma AG, Lichtstrasse 35, Basel, CH, Switzerland; 8 Novartis Ireland Limited, Dublin, Ireland; 9 The Alan Turing Institute, London, UK; 10 Radcliffe Department of Medicine, University of Oxford, Oxford, UK; 11 Novartis Pharmaceuticals Corporation, East Hanover, NJ, USA; 12 Novartis Institutes for BioMedical Research, Basel, CH, Switzerland; 13 Novartis Pharma AG, Global Drug Development, Basel, CH, Switzerland

**Keywords:** genome-wide association studies, inflammatory diseases, rheumatic diseases, biological therapy, clinical trials

## Abstract

Response to the anti-IL17 monoclonal antibody secukinumab is heterogeneous, and not all participants respond to treatment. Understanding whether this heterogeneity is driven by genetic variation is a key aim of pharmacogenetics and could influence precision medicine approaches in inflammatory diseases. Using changes in disease activity scores across 5,218 genotyped individuals from 19 clinical trials across four indications (psoriatic arthritis, psoriasis, ankylosing spondylitis, and rheumatoid arthritis), we tested whether genetics predicted response to secukinumab. We did not find any evidence of association between treatment response and common variants, imputed HLA alleles, polygenic risk scores of disease susceptibility, or cross-disease components of shared genetic risk. This suggests that anti-IL17 therapy is equally effective regardless of an individual’s genetic background, a finding that has important implications for future genetic studies of biological therapy response in inflammatory diseases.

## Teaser

We investigated the role of genetics in clinical response to secukinumab in 5,218 individuals from 19 clinical trials across four indications. We found no association between response and common variants, imputed HLA alleles, polygenic disease susceptibility risk, or cross-disease shared genetic risk. Anti-IL17 therapy appears equally effective regardless of genetics.

## Main text

Response to biological therapy in inflammatory disease is typically heterogeneous, and even in highly successful and widely used treatments (such as tumor necrosis factor inhibitor [TNFi] therapies), a significant fraction of participants fail to respond to treatment.^1^ A common hypothesis is that heterogeneity in response reflects genetic differences between individuals or, relatedly, genetically distinct disease subtypes with different molecular etiologies.^2^ If this hypothesis is true and genetic biomarkers for drug response can be identified, this could lead to new understanding of the biology of drug response or discovery of biomarkers to stratify subgroups of participants to specific treatments to increase response rates.^3^^,^^4^^,^^5^

A further hypothesis is that genetic pathways that place individuals at risk of disease may also influence their response to therapy, and this may be captured by polygenic scores of disease susceptibility.^6^^,^^7^ This is even more plausible where the pathway being treated is known to play an important role in disease susceptibility, as for anti-interleukin-17 (IL17) and anti-IL23 therapies.^8^^,^^9^ When multiple indications are treated by the same drug, the same risk variants may have differing directions of effect for related diseases.^10^ To overcome this pleiotropy of inflammatory pathways, genetic risk can be modeled across multiple inflammatory diseases using several orthogonal genetic components^11^ that may correlate with treatment response.

Multiple genome-wide association studies (GWASs) of response to biological therapies have been published.^12^^,^^13^^,^^14^^,^^15^ These primarily consist of post-approval studies of drugs in regular use with most studying TNFi therapies. The most consistent association known is between HLA-DQA1^∗^05 alleles and development of anti-drug antibodies.^14^^,^^15^^,^^16^ However, relatively low sample sizes, small number of associations, and lack of consistent cross-replication of effects between these studies has made it difficult to draw firm conclusions of the role of genetics in treatment response. In addition, these studies included only participants under active treatment and thus could not discriminate between prognostic biomarkers (which correlate with outcomes independently of treatment) and predictive biomarkers (which correlate with response to a specific treatment).^17^ In this study, we used data from clinical trial participants, randomized to secukinumab or placebo, to test for predictive biomarkers.

Secukinumab (brand name Cosentyx) is a widely used therapy for treating inflammatory diseases by blocking the pro-inflammatory IL-17 signaling pathway. It has been approved for use in plaque psoriasis (Pso), ankylosing spondylitis (AS), psoriatic arthritis (PsA), and other inflammatory conditions and has been extensively studied in clinical trials (including in indications for which it has not been approved, such as rheumatoid arthritis [RA]). Individual participants’ responses to treatment are heterogeneous within indications, with response rates of 81.6% for Pso,^18^ 62.6% for PsA,^19^ 60.5% for AS,^20^ and 30.7% for RA.^21^ Even for the same disease and outcome, there can be substantial variability in response rates from study to study in real-world settings,^22^ and there is some evidence that response rates can vary between countries, sexes, and disease subtypes.^23^^,^^24^

There have, to date, been no genome-wide studies of response to anti-IL17 therapy, though two small studies in psoriasis have investigated candidate genes, primarily within the human leukocyte antigen (HLA) locus.^23^^,^^25^^,^^26^ The clinically significant but heterogeneous response to a biologic therapy, measured in many clinical trials, provides a unique dataset to test for predictive biomarkers. Here, we tested whether individual genetic variants (including SNPs and HLA alleles), polygenic risk scores (PRSs) of susceptibility, or components of genetic risk are associated with response to anti-IL17 therapy in a large dataset from multiple randomized placebo-controlled trials across four diseases.

We gathered genetic and clinical data from 19 clinical trials of anti-IL17 therapy with secukinumab (Table S1) across four indications (PsA, RA, AS, and Pso). We used continuous measures of disease activity as our primary outcomes: the disease activity score 28 with C-reactive protein (DAS28-CRP) for PsA and RA,^27^ the AS disease activity score with CRP (ASDAS-CRP) for AS,^28^ and the psoriasis area and severity index (PASI) score for Pso.^29^ After quality control, we had complete clinical and genotype data on 5,218 participants, including 4,063 treated with anti-IL17 therapy and 1,151 placebo controls. Demographic and clinical characteristics of these participants are shown in Table 1 and are broken down by treatment, randomization status, and exclusion status in Table S2.

**Table 1:** Clinical and demographic statistics of participants included in this study, broken down by indication; treated cases include any participants randomized to receive secukinumab, regardless of dosing regimen

	**AS**	**PsA**	**Pso**	**RA**
	**(n = 754)**	**(n = 2,006)**	**(n = 1,636)**	**(n = 822)**
Primary outcome	ASDAS-CRP	DAS28-CRP	PASI	DAS28-CRP
Baseline value of primary outcome (median [min, max])	3.63 [0.528, 6.22]	4.59 [1.37, 8.28]	4.59 [1.37, 8.28]	5.72 [2.58, 8.01]
Delta in primary outcome (median [min, max])	−0.866 [−4.62, 1.84]	−1.05 [−5.25, 2.49]	−1.05 [−5.25, 2.49]	−1.22 [−5.58, 3.03]
# of studies	4	6	4	5
# of treated cases	506	1,483	1,495	583
# of placebo cases	248	523	141	239
Age (median [min, max])	42.0 [17.0, 82.0]	50.0 [16.0, 83.0]	45.0 [15.0, 87.0]	55.0 [19.0, 84.0]
Sex				
Female	249 (33.0%)	1,029 (51.3%)	507 (31.0%)	661 (80.4%)
BMI category				
Underweight	16 (2.1%)	9 (0.4%)	15 (0.9%)	33 (4.0%)
Normal weight	256 (34.0%)	459 (22.9%)	374 (22.9%)	308 (37.5%)
Overweight	213 (28.2%)	681 (33.9%)	576 (35.2%)	239 (29.1%)
Obese	269 (35.7%)	857 (42.7%)	671 (41.0%)	242 (29.4%)
Methotrexate use at recruitment				
Yes	87 (11.5%)	876 (43.7%)	not used	741 (90.1%)
Previous anti-TNF treatment failure at recruitment				
Yes	530 (70.3%)	1,517 (75.6%)	not used	not used
PC ancestry				
AMR	117 (15.5%)	200 (10.0%)	229 (14.0%)	193 (23.5%)
EAS	<5 (<0.6%)	17 (0.8%)	77 (4.7%)	110 (13.4%)
EUR	632 (83.8%)	1,741 (86.8%)	1,234 (75.4%)	499 (60.7%)
SAS	<5 (<0.6%)	48 (2.4%)	96 (5.9%)	17 (2.1%)
AFR	<5 (<0.6%)	–	–	<5 (<0.4%)

Delta is the change in primary outcome between baseline and assessment week (week 12 for Pso, week 16 for all other indications). AS, ankylosing spondylitis; PsA, psoriatic arthritis; Pso, psoriasis; RA, rheumatoid arthritis; BMI, body mass index; PC ancestry, ancestry assigned by principal component analysis; AMR, admixed American; EAS, east Asian; EUR, European; SAS, south Asian; AFR, African.

Power calculations showed that these samples sizes provide enough power to detect common variants or risk scores that exert a clinically meaningful effect. For the best-powered indication (PsA, n = 2,006), our primary analysis had >80% power to detect a common variant that increases the difference between DAS28-CRP in treatment vs. controls by 0.06 units per allele (Figure S1), equivalent to an 8% modification in the effect of treatment.

For our primary analyses, we carried out a genome-wide treatment-by-genotype interaction study for each of the four indications separately. We stratified participants by their predicted continental ancestry (PC ancestry, defined based on their distance from nearest continental population on a principal component analysis [PCA] of samples from the 1000 Genomes Project^30^) and meta-analyzed the results across ancestry groups (after removing ancestry groups with low sample size). We included three genetic principal components as well as known demographics and other confounders in the regression model (see supplemental methods for details). We detected evidence of miscalibration in test statistics in the quantile-quantile (Q-Q) plots for two of these analyses (Pso PASI and RA DAS28-CRP, Figure S2) due to model misspecification driven by heteroscedasticity, a known problem with gene-treatment interaction models.^31^^,^^32^ We thus replaced these two analyses with robust regression analyses^33^ (see supplemental methods for details).

**Figure 1:**
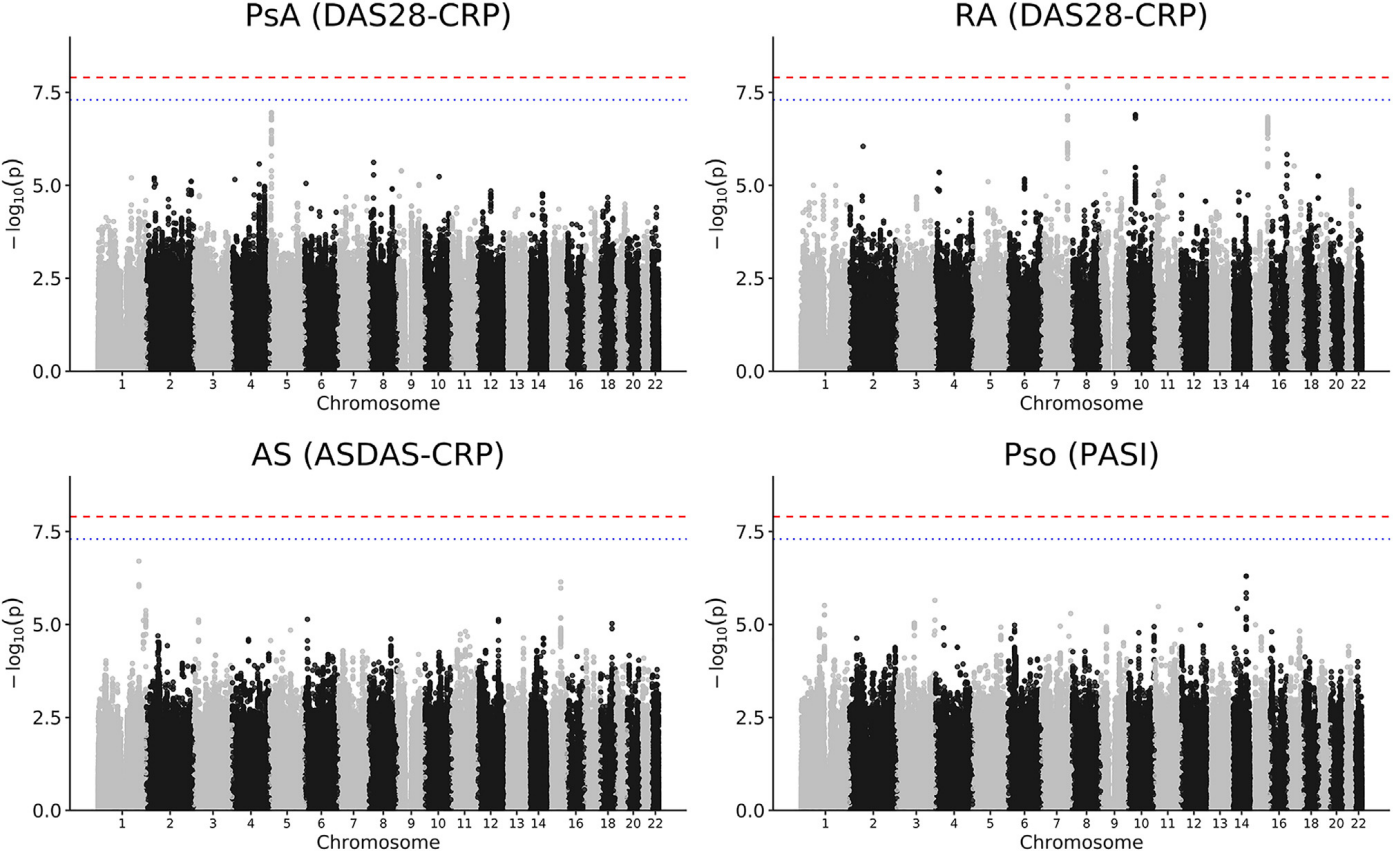
Manhattan plots of treatment-by-genotype interaction GWASs for the primary outcome of each of the four indications tested p values are from linear regression. The blue line shows genome-wide significance (p = 5 × 10^−^^8^) and the red line shows analysis-wide significance controlling for four indications (p = 1.25 × 10^−^^8^). Each plot shows a meta-analysis of all ethnicities with a sample size of at least 100.

Linkage disequilibrium score (LDSC^34^^,^^35^) regression did not show a significant treatment-by-genome heritability term, though confidence intervals were large (Table S3). No variant passed study-wide significance correcting for four tests (p < 1.25 × 10^−8^) (Figure 1). We also tested variants previously associated with response to other biologic therapies and did not find any significant variant-treatment interactions after correcting for multiple testing (Table S4). One variant, rs11762062, met genome-wide but not study-wide significance for DAS28-CRP in RA (p = 2.08 x 10^−8^, Table S5), but this variant did not replicate in the PsA DAS28-CRP analysis (p = 0.332).

**Figure 2:**
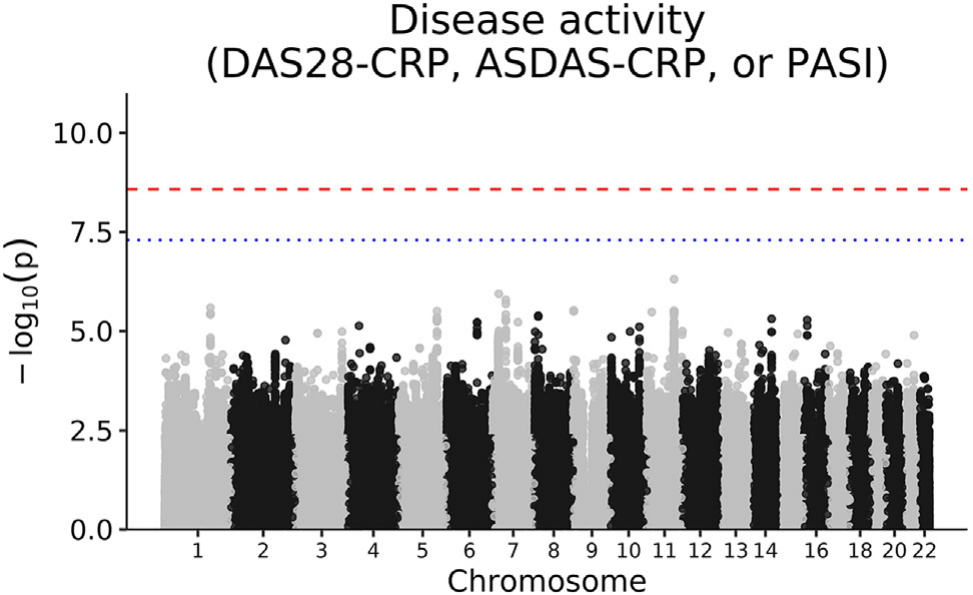
Manhattan plots of cross-indication meta-analyses of treatment-by-genotype interaction GWASs for primary (disease activity) outcomes p values are from linear regression. The blue line shows genome-wide significance (p = 5 × 10^−^^8^) and the red line shows analysis-wide significance controlling for 19 analyses (p = 2.63 × 10^−^^9^, i.e., correcting for all individual GWASs and GWAS meta-analyses).

To maximize power, we also carried out a cross-indication meta-analysis of these results. We meta-analyzed the primary outcomes using a sample-size weighted *Z* score meta-analysis in order to make less strict assumptions about comparability of the scale of effects across indications. We did not find any study-wide or genome-wide significant associations (Figure 2). The sample sizes in this case were large enough to make inferences using the LDSC heritability analysis Table S3), which showed that the variability in disease activity explained by genotype-treatment interactions, when averaged across indications, must be relatively small (upper bound of 28%).

**Figure 3:**
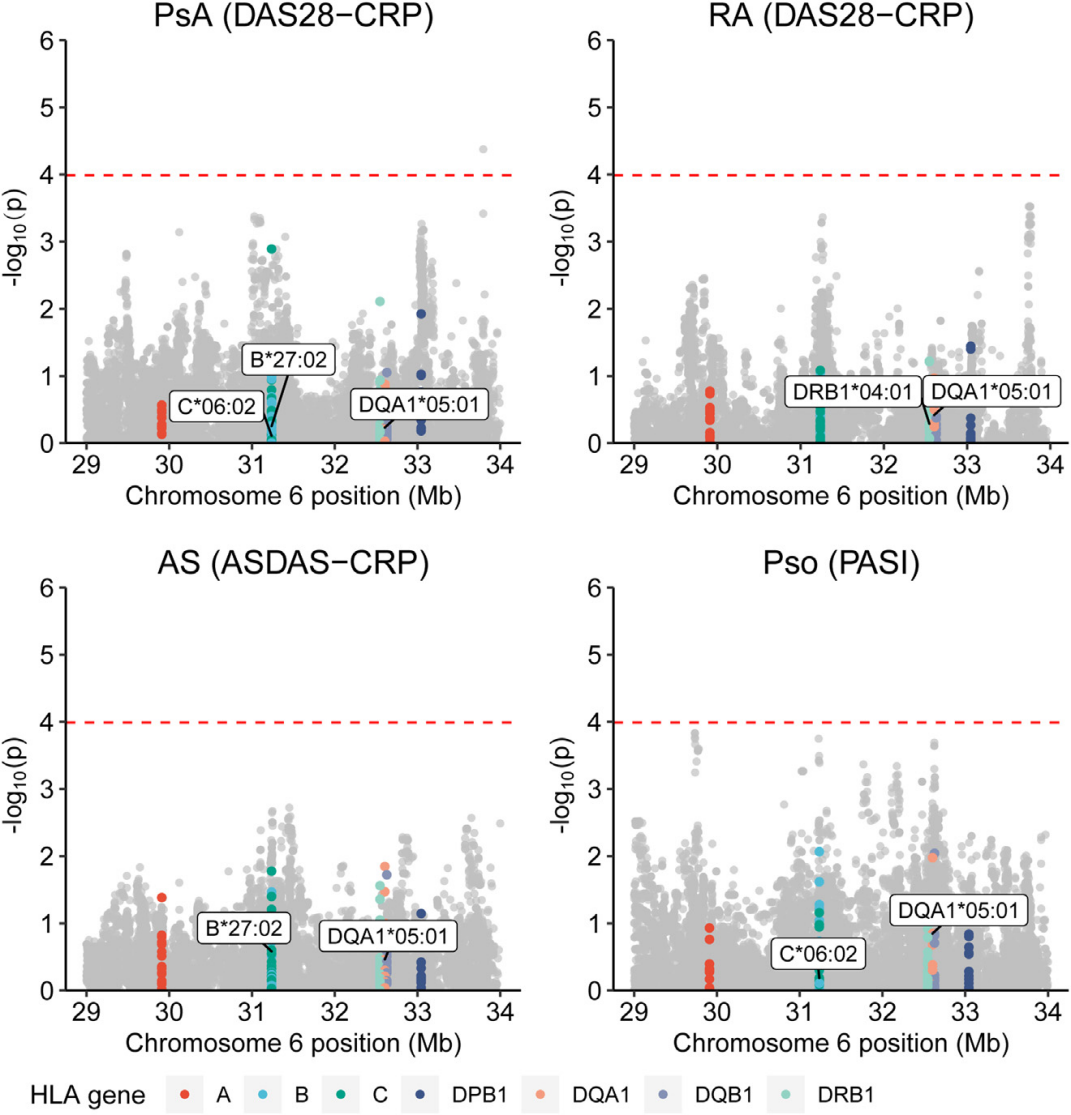
Regional plots of treatment-by-genotype interaction analysis for SNPs and HLA alleles in the HLA region for the primary outcome of each of the four indications tested p values are from linear regression. Gray dots are SNPs and colored dotted are HLA alleles. The red dashed line shows analysis-wide significance for HLA alleles (p = 1.02 × 10^−^^4^), correcting for 489 tests of HLA alleles in four indications. Major susceptibility alleles for each indication are highlighted. Mb, Megabases.

Next, we tested for interactions between drug response and imputed HLA alleles. We did not find any significant associations after adjusting for the 489 allele/outcome combinations tested (Figure 3; Table S6) and did not find any nominally significant associations with any of the major risk HLA alleles for the four indications, with the HLA-Cw6 (C^∗^06) allele previously associated with response to anti-IL17 therapy^26^ or with the HLA-DQA1^∗^05 alleles previously associated with TNFi immunogenicity^14^^,^^15^^,^^16^ (Table S4).

**Figure 4:**
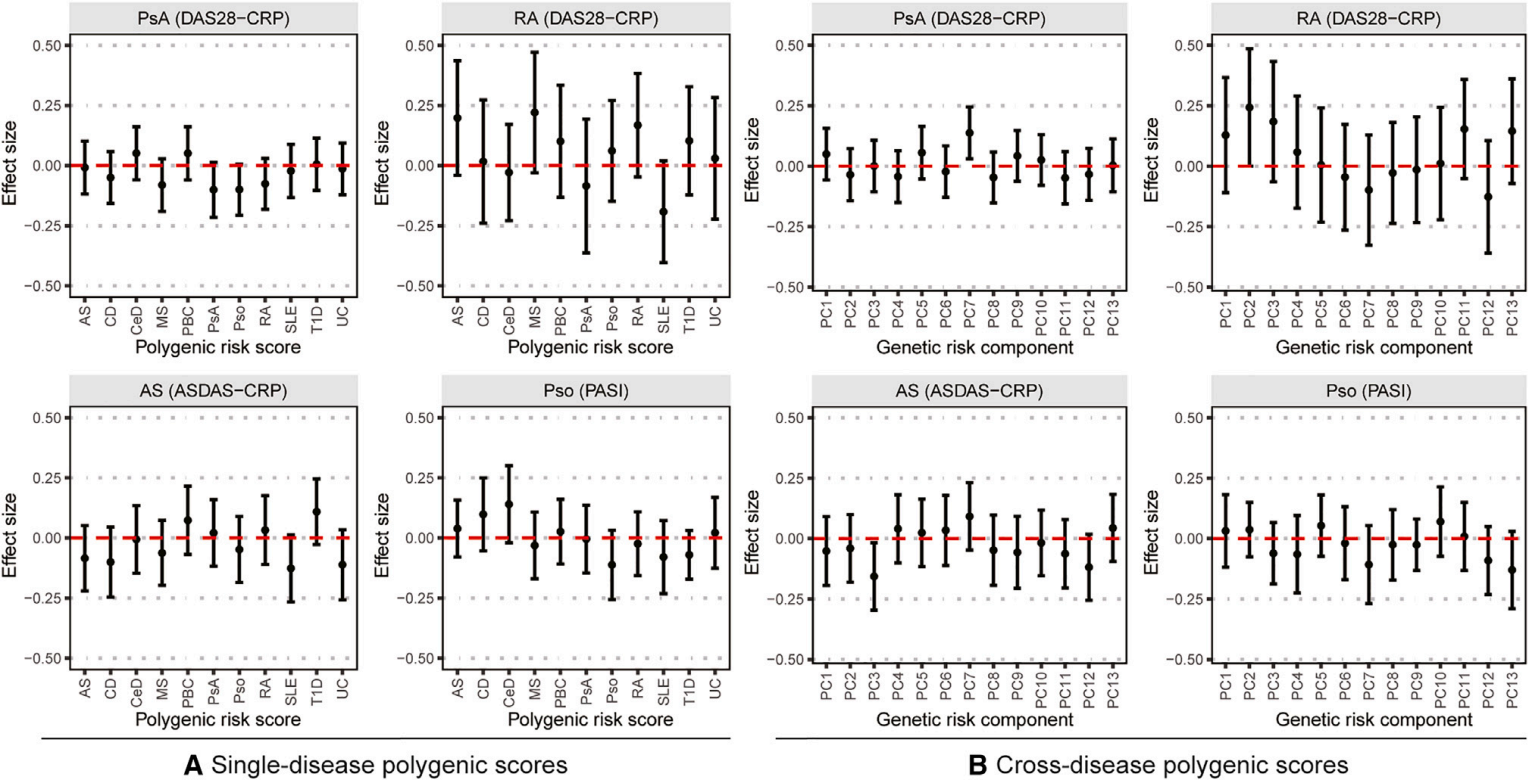
Interaction effect size between treatment and single disease or cross-disease polygenic risk scores on primary outcomes for the four indications Effect sizes are given per standard deviation of the polygenic score. Error bars are 95% confidence intervals. Red dashed line corresponds to effect size = 0.

To test the hypothesis that disease-susceptibility variants could influence drug response, we also calculated PRSs for 11 inflammatory diseases, including the four indications and seven other related diseases (celiac disease, Crohn disease, multiple sclerosis, primary biliary cirrhosis, systemic lupus erythematosus, type 1 diabetes, and ulcerative colitis; see Table S7 for references). In addition, we calculated risk scores based on shared orthogonal genetic components of immune and inflammatory disease risk following Burren et al.^11^ However, none of these risk scores predicted response to anti-IL17 therapy (Figure 4; Tables S8 and S9).

Our primary analysis used clinical disease activity scores, which are, in most cases, composites of multiple measures, including numerical ratings of clinician- or patient-assessed disease state, objective measures of disease symptoms (such as swollen joint counts), and biomarker measures. We also ran 12 further genome-wide treatment-by-genotype interaction analyses for a range of secondary outcomes, including laboratory-based and self-reported measures (see supplemental methods for a full list and Figures S3–S7 for results; note that one outcome, PsA ACRn (percentage improvement in American College of Rheumatology multidimensional response score), was replaced with a robust regression analysis). We did not see any study-wide significant associated variants correcting for 16 (4 primary and 12 secondary) analyses (p < 3.12 × 10^−9^), and none of the disease/endpoint pairs showed a Bonferroni-corrected significant treatment-by-genome heritability component in the LDSC analysis (Table S3). We also carried out HLA and PRS analyses with secondary outcomes, none of which showed significant associations (Figures S8–S10). We meta-analyzed CRP and erythrocyte sedimentation rate (ESR) across indications and did not find any study-wide significant associations (Figures S11 and S12) or appreciable heritability (Table S3).

The aim of this paper was to test for predictive, not prognostic, effects of genetics on drug response. However, to maximize power, we also carried out a joint predictive-prognostic analysis using a joint test^36^ (i.e., a combined test for either a main effect of genotype on outcome regardless of treatment status and an interaction effect with treatment). This did not produce any study-wide significant results, and three genome-wide but not study-wide significant results are presented in Table S10. We did find that a previously reported SNP (rs7195994) association with prognostic response to TNFi at *FTO*^13^ replicated at nominal significance (p < 0.05) in our prognostic main effect analysis (Table S4).

Based on our findings, we can rule out several important hypotheses about the genetics of response to anti-IL17 therapy. We have shown that common variants do not have a moderate-to-large effect in response to anti-IL17 therapy, which includes common SNPs and also, more surprisingly, common HLA alleles. We can also exclude a large effect on response from the genetic pathways that influence susceptibility, either measured by PRSs for susceptibility to individual inflammatory diseases or by scores that reflect shared genetic pathways across inflammatory diseases. This adds strong evidence to support the claim that genetic heterogeneity is not a large driver of response to anti-IL17 therapy and that secukinumab is equally effective regardless of genetic background.

There are some limitations to our study that mean certain scenarios cannot be ruled out. Our use of genotyping arrays rather than genome sequencing meant we were unable to detect rare variants, so it is possible that lower-frequency variants of large effect may predict drug response. In addition, the wide confidence intervals on our SNP heritability estimates mean that we cannot rule out a moderate polygenic component consisting of a large number of variants of very small effect. Given our analysis of both single-disease and shared PRSs, if a polygenic component of drug response does exist, we posit that it would be uncorrelated with the genetics of disease susceptibility. This has been noted in other contexts, including the observation that genetic predictors of elevated low-density lipoprotein (LDL) capture the prognostic but not predictive response to LDL-lowering treatment and has motivated the development of direct pharmacogenomic PRS (PGx PRS) techniques.^37^ Our study was also limited by the outcomes collected, as past research has found that both disease activity and biomarker data are only incompletely correlated with imaging-based outcomes.^38^ It remains possible that genetics may influence components of disease response not well captured by traditional clinical trial outcomes. Our sample size was also limited for some indications, so while we had high power for some analyses (particularly PsA and cross-indication outcomes), highly disease-specific gene-by-treatment interactions could have been missed for some indications (AS and RA, where N < 1000). Finally, some of our indications showed test-statistic inflation (as discussed in supplemental methods), which we attribute to model misspecification due to heteroscedasticity and to low sample size and minor allele frequency in certain smaller PC ancestry groups. Filtering and changes in model choice largely removed this inflation; however, it remains possible that some polygenic signal may also contribute to this inflation signal.

Secukinumab directly targets a pathway that has been repeatedly implicated in the genetics of inflammatory diseases,^39^^,^^40^^,^^41^^,^^42^^,^^43^ including risk variants in genes both upstream of IL-17 production (*IL23R*, *TYK2*, *JAK2*, *STAT3*) and downstream of IL-17 response (*TRAF3IP2*/ACT1, *TNFAIP3*/A20). While genetic variation in this pathway impacts disease susceptibility, this variation does not seem to significantly influence response to treatment of that same pathogenic pathway. This may also have implications for hypotheses about endotypes in inflammatory disease, as genetically distinct participant subgroups with a strong differential response to treatment would manifest as genetic predictors of disease response in our study.

We believe that these results have important implications for future genetic studies of biological therapy in inflammatory disease and for the field of pharmacogenetics. Secukinumab has a large treatment effect while still maintaining significant unexplained variation in response, and this study was well powered and covered a variety of indications, outcomes, and ancestry groups. This suggests that, even in favorable conditions, finding predictive genetic biomarkers of response can prove difficult. While we do not yet know how widely this generalizes to other treatments and pathways, we should consider the possibility that genetics may not play a major role in response to other biological therapies as well, or that much larger sample sizes than can be acquired in even large clinical trials will be required to map small effect variants. Large cohorts of real-world participants undergoing routine treatment, such as the IBD BioResource^44^ or HIPPOCRATES consortium,^45^ may provide sample sizes required for mapping such variants, though these raise caveats around the lower precision of real-world outcomes and lack of randomized placebo control in real-world studies. It is likely that leveraging the advantages of both clinical trial and real-world data will be required to finally map the genetics of response to biological therapy while distinguishing predictive and prognostic biomarkers. We hope that this study will be a starting point for larger meta-analyses of the genetics of treatment response for inflammatory and autoimmune diseases.

## Data Availability

Anonymized clinical trial data are available upon request through Novartis’ voluntary data-sharing process on ClinicalStudyDataRequest.com. Inquiries to access subject-level genetic data can be made through ClinicalStudyDataRequest.com and require a Sponsor Data-Sharing Agreement with Novartis Pharma AG. Full genome-wide summary statistics are available on the EBI GWAS Catalog (https://www.ebi.ac.uk/gwas/, accession numbers GCST90274734 to GCST90274752).

## Supplemental information

Data S1. Tables S1–S11

Document S2. Article plus supplemental information
