# Supplementary material for: Response to anti-IL17 therapy in inflammatory disease is not strongly impacted by genetic background
Source: Am J Hum Genet. 2023 Sep 1;110(10):1817–24. doi: 10.1016/j.ajhg.2023.08.010 (PMC10577077; doi:10.1016/j.ajhg.2023.08.010)
Supplement: Document S1. Figures S1–S13 and supplemental methods [file mmc1.pdf]

The American Journal of Human Genetics, Volume *110*

Supplemental information

Response to anti-IL17 therapy in inflammatory disease is not strongly impacted by genetic background

Cong Zhang, Konstantin Shestopaloff, Benjamin Hollis, Chun Hei Kwok, Claudia Hon, Nicole Hartmann, Chengeng Tian, Magdalena Wozniak, Luis Santos, Dominique West, Stephen Gardiner, Ann-Marie Mallon, Aimee Readie, Ruvie Martin, Thomas Nichols, Michael T. Beste, Jonas Zierer, Enrico Ferrero, Marc Vandemeulebroecke, and Luke Jostins-Dean

Supplemental Figures

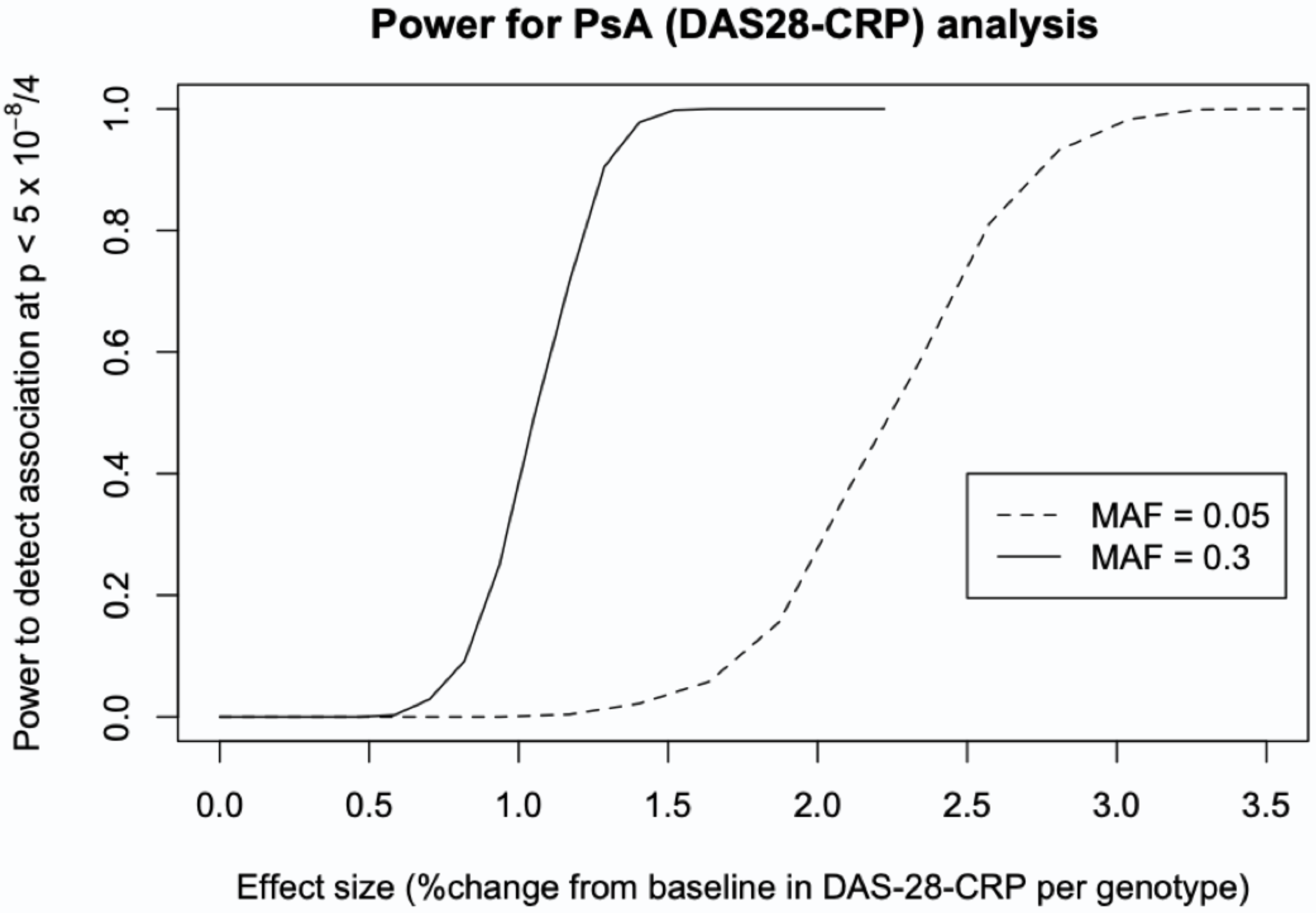

Figure S1: Power curves for a genome-wide genotype-by-treatment interaction for the largest indication/endpoint combination (PsA DAS28-CRP), for a lower frequency (MAF = 5%) and higher frequency (MAF = 30%) variant. MAF = Minor Allele Frequency.

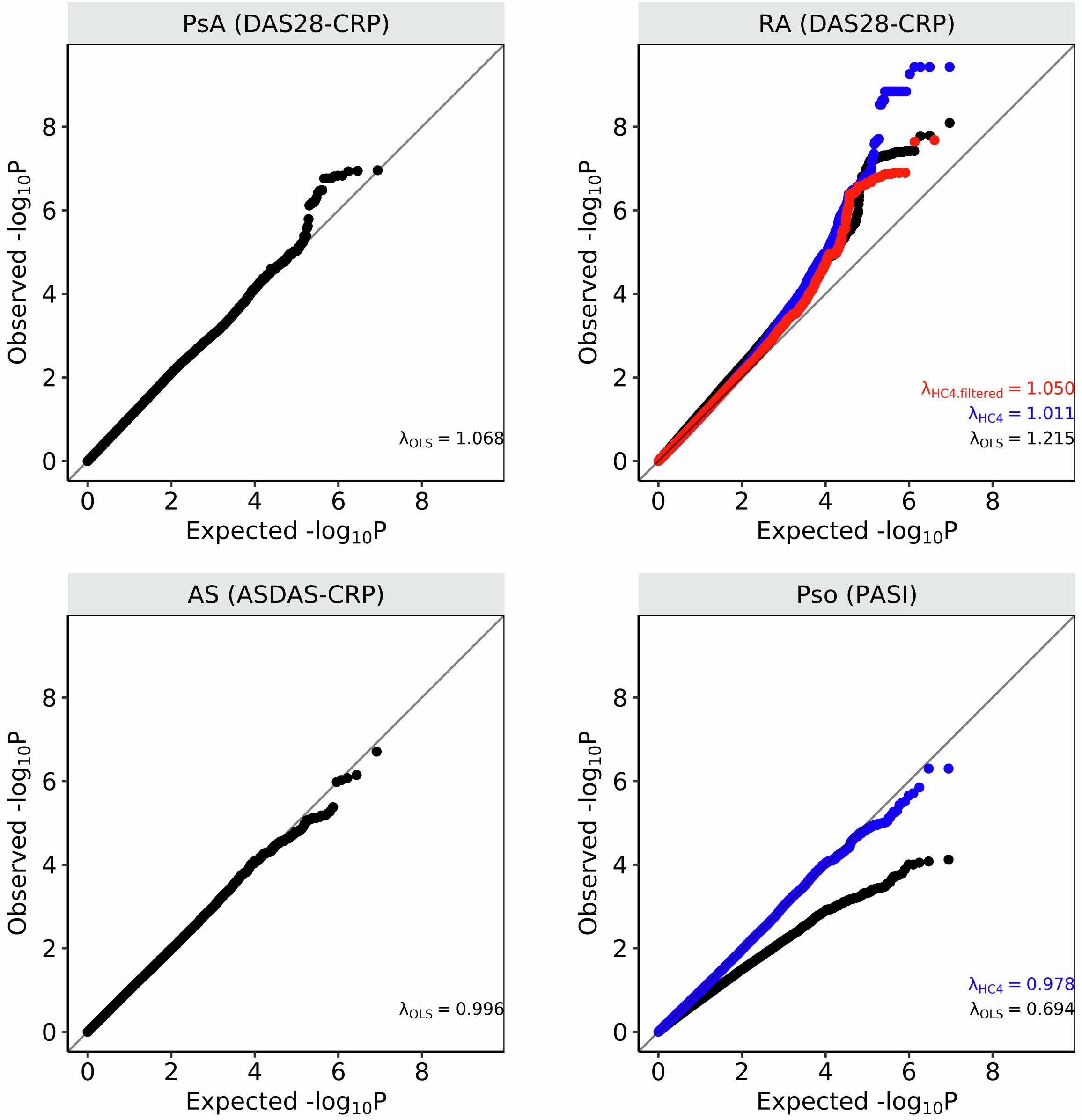

Figure S2: QQ plots for the treatment-by—genotype interaction GWAS for each of the primary outcomes for the four indications. Black dots shows the OLS regression result. The coloured dots show analyses that were rejected due to inflated or deflated QQ plots (blue for robust regression result with HC4 estimator used for rejected OLS regression, red for filtered robust regression result with HC4 estimator used for rejected robust regression). The OLS regression for PsA and AS, the robust regression for Pso and the heterogeneity-filtered robust regression for RA were used in the paper.

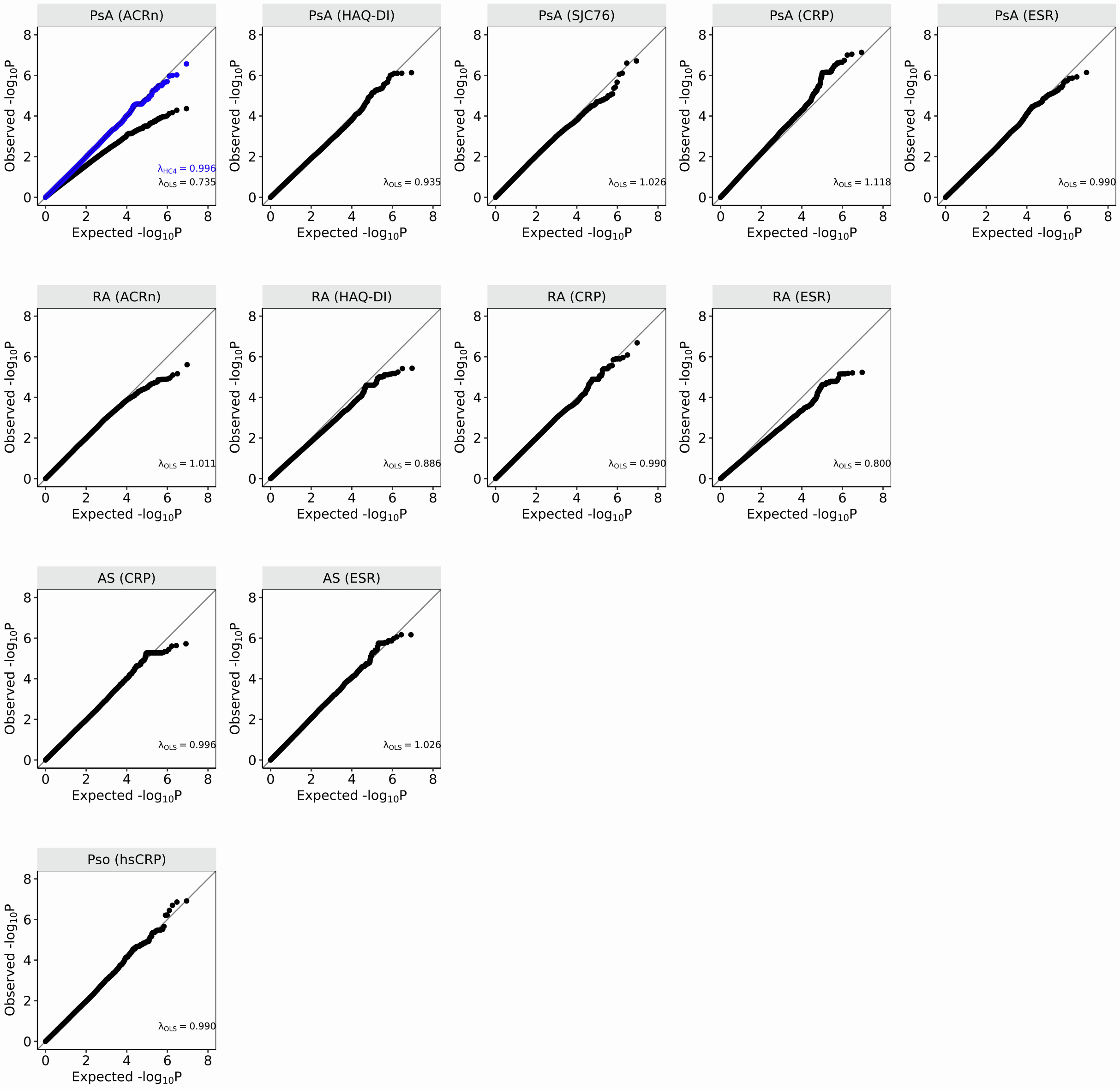

Figure S3: QQ plots for the treatment-by-genotype interaction GWAS for each of the secondary outcomes for the four indications. Black dots shows the OLS regression result. The coloured dots show analyses that were rejected due to inflated or deflated QQ plots (blue for robust regression result with HC4 estimator used for rejected OLS regression). The robust regression for PsA ACRn, OLS regression for all other outcomes were used in the paper.

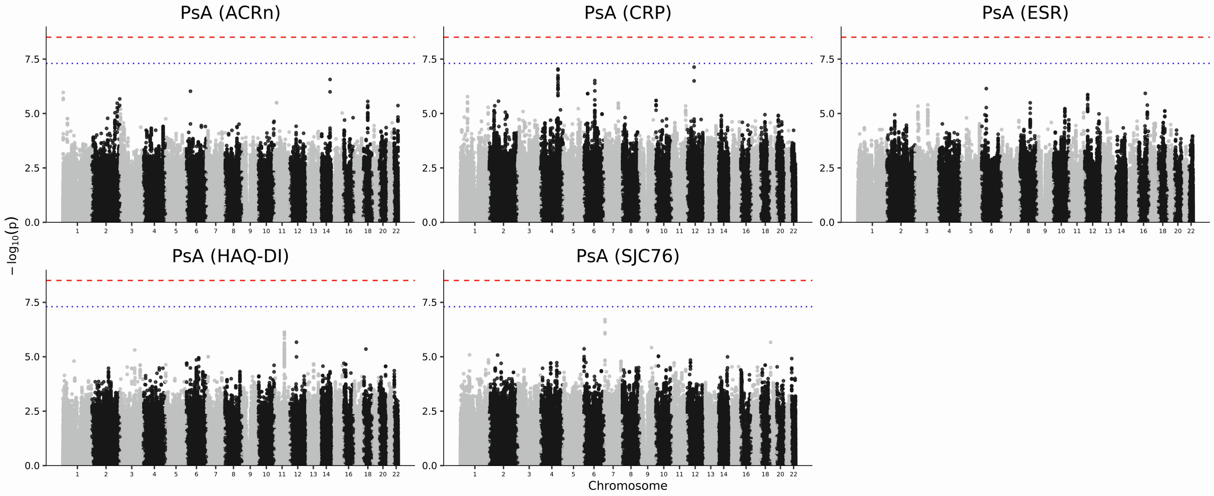

Figure S4: Manhattan plots of treatment-by-genotype interaction GWAS for the secondary outcomes for PsA. The blue line shows genome-wide significance (p = 5e-8) and the red line shows analysis-wide significance controlling for 16 analyses across all four indications (p = 3.12 x 10^{-9}).

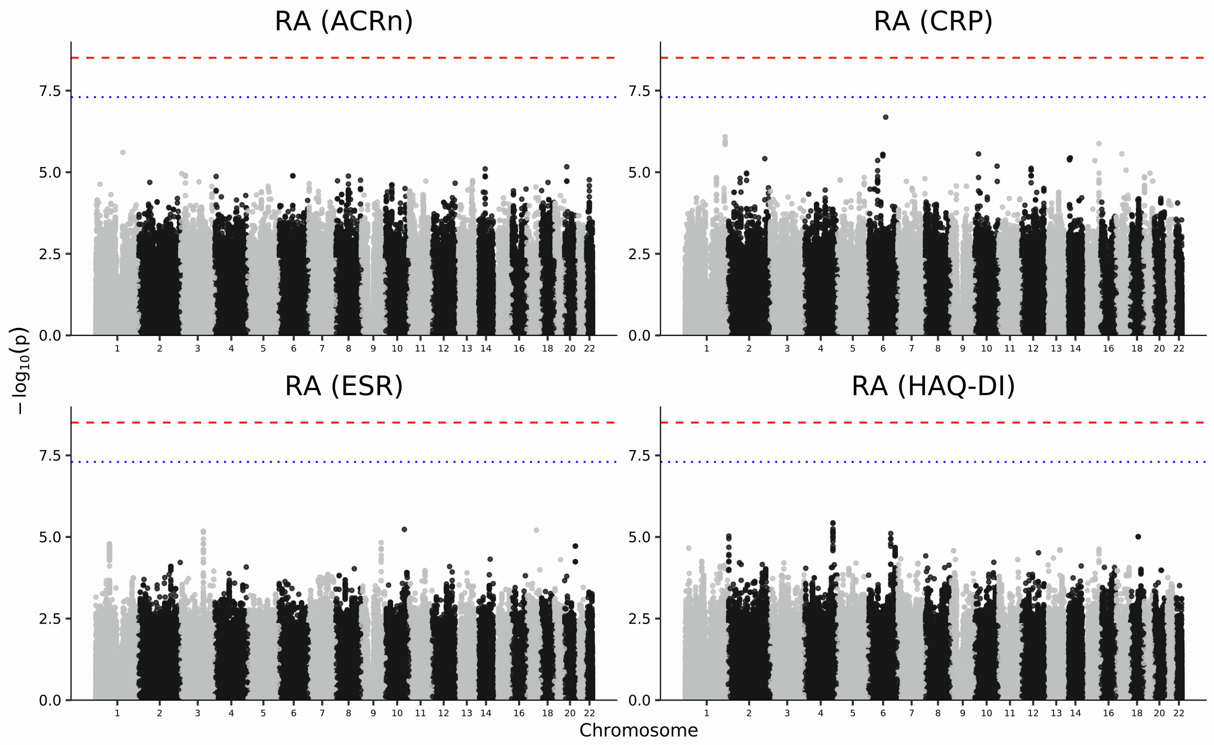

Figure S5: Manhattan plots of treatment-by-genotype interaction GWAS for the secondary outcomes for RA. The blue line shows genome-wide significance ($p = 5 \times 10^{-8}$) and the red line shows analysis-wide significance controlling for 16 analyses across all four indications ($p = 3.12 \times 10^{-9}$).

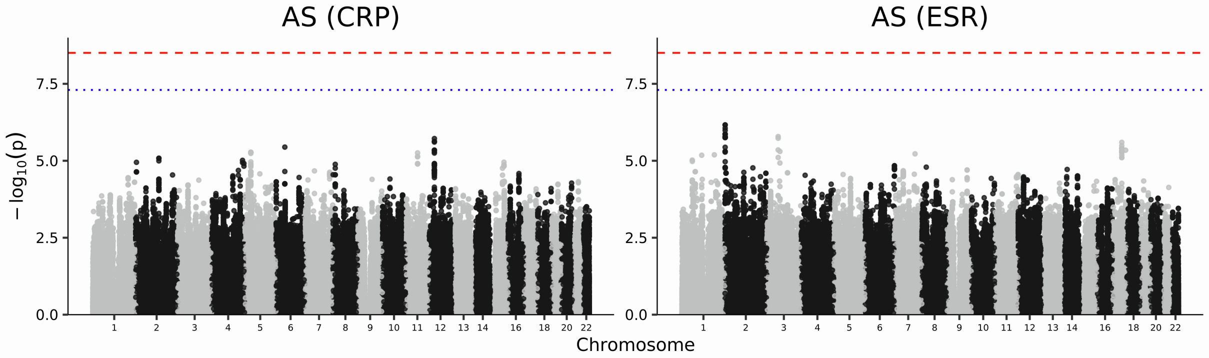

Figure S6: Manhattan plots of treatment-by-genotype interaction GWAS for the secondary outcomes for AS. The blue line shows genome-wide significance ($p = 5 \times 10^{-8}$) and the red line shows analysis-wide significance controlling for 16 analyses across all four indications ($p = 3.12 \times 10^{-9}$).

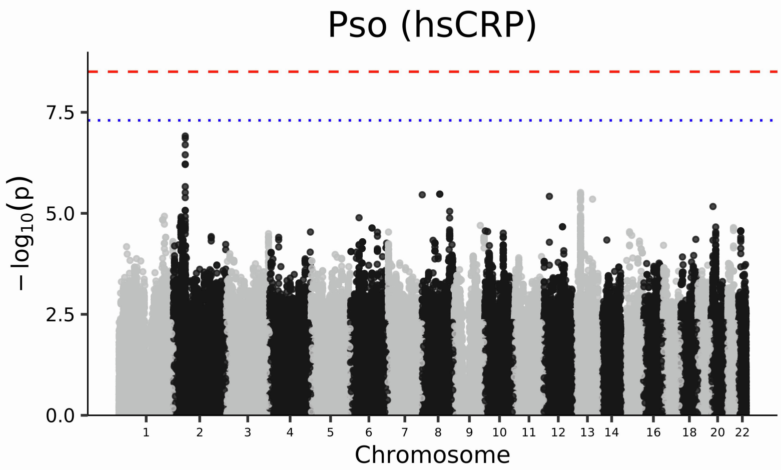

Figure S7: Manhattan plots of treatment-by-genotype interaction GWAS for the secondary outcomes for Pso. The blue line shows genome-wide significance ($p = 5 \times 10^{-8}$) and the red line shows analysis-wide significance controlling for 16 analyses across all four indications ($p = 3.12 \times 10^{-9}$).

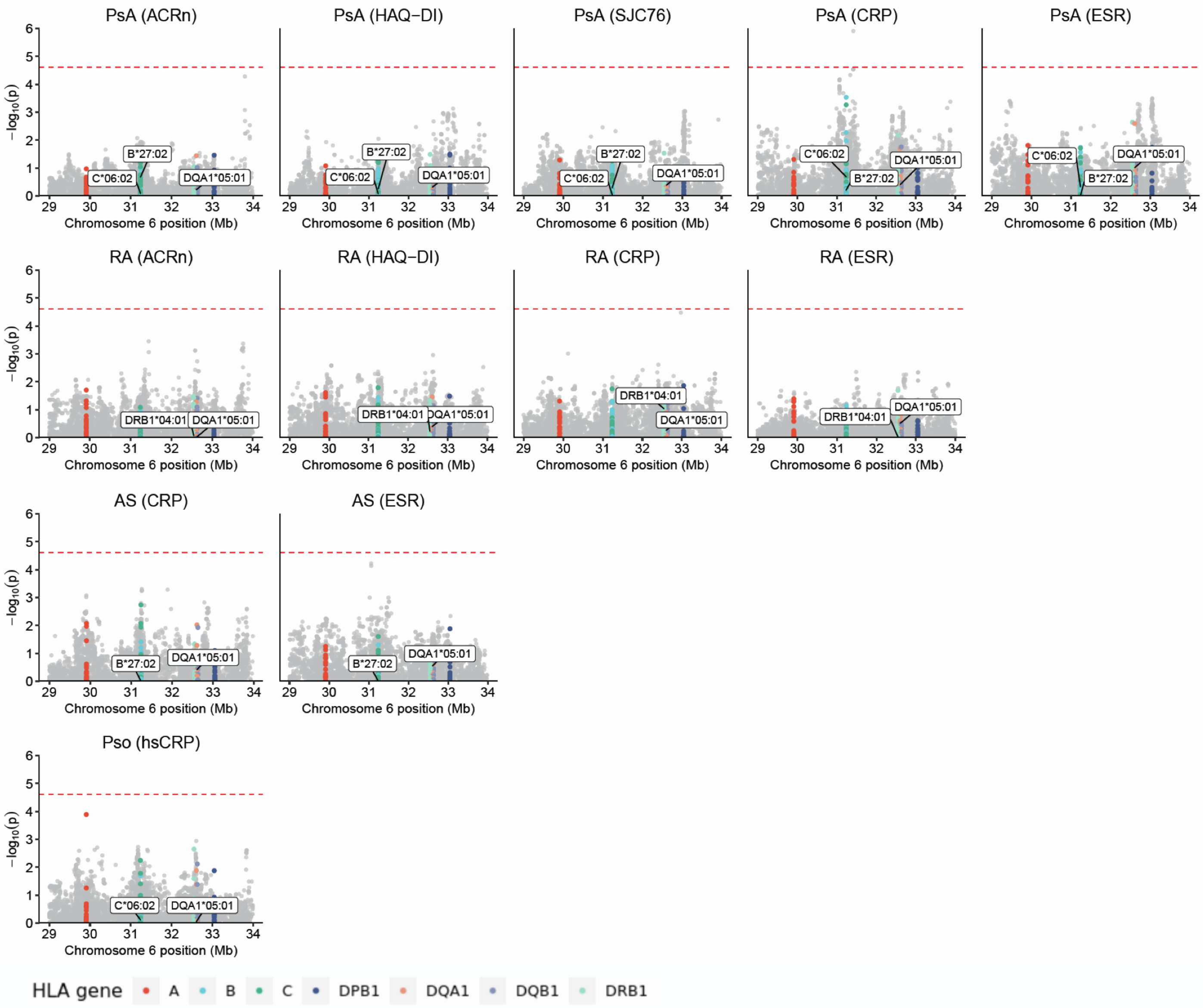

Figure S8: Regional plots of treatment-by-genotype interaction analysis for SNPs and HLA alleles in the HLA region for the secondary outcomes of each of the four indications tested (PsA, RA, AS and Pso). Grey dots are SNPs, and coloured dotted are HLA alleles. The red dashed line shows analysis-wide significance for HLA alleles ($p = 2.41 \times 10^{-5}$), correcting for a total of 2,074 tests of HLA alleles in 15 endpoints. Mb = Megabases.

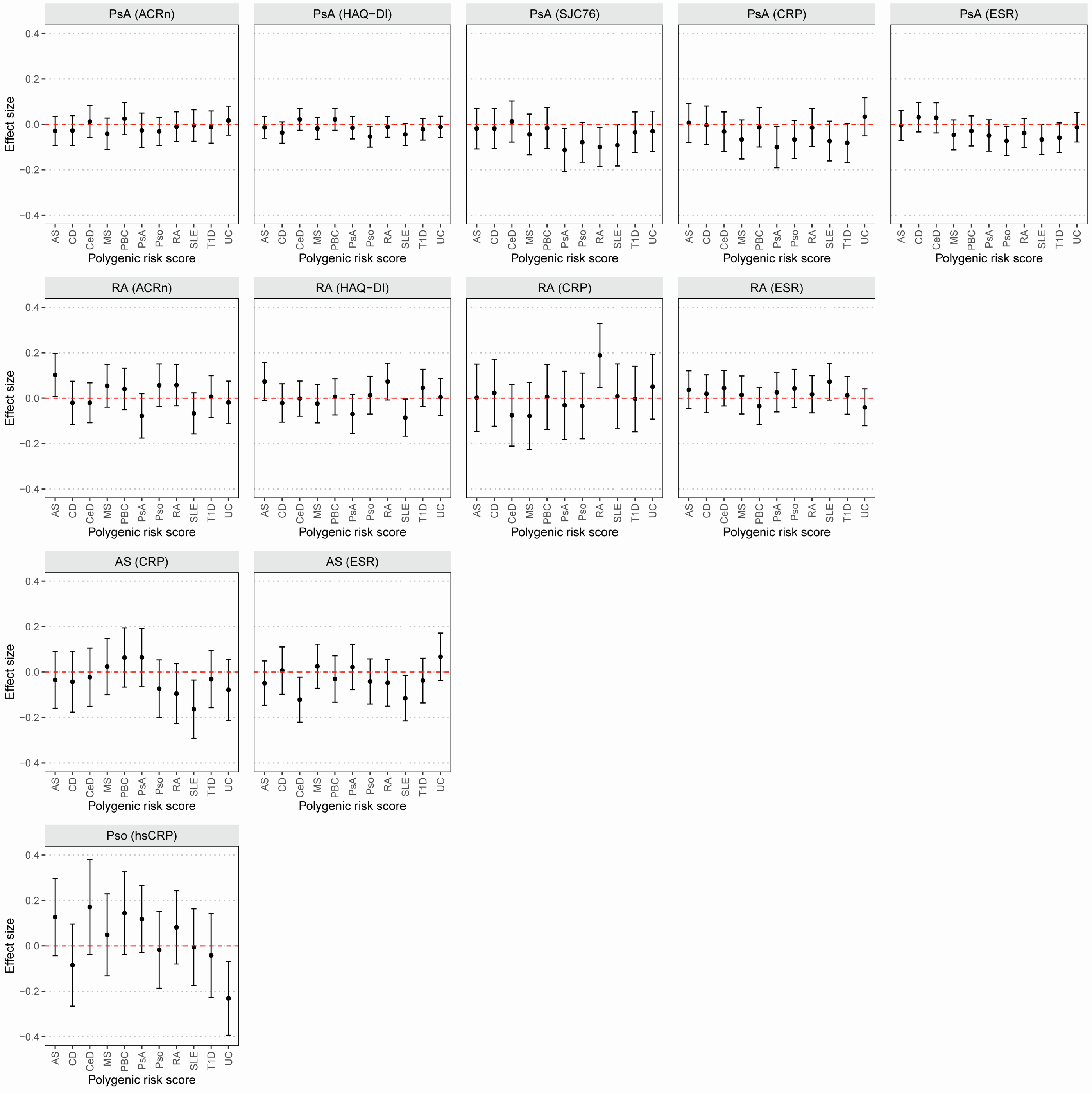

Figure S9: Interaction effect size between treatment and single disease polygenic risk scores on secondary outcomes for four indications. Effect sizes are given per standard deviation of the polygenic score. Error bars are 95% confidence intervals. Red dashed line corresponds to effect size = 0. Abbreviation of polygenic risk score traits: Ankylosing Spondylitis (AS), Celiac Disease (CeD), Crohn's Disease (CD), Multiple Sclerosis (MS), Primary Biliary Cirrhosis (PBC), Psoriatic Arthritis (PsA), Rheumatoid Arthritis (RA) Systemic Lupus Erythematosus (SLE), Type 1 Diabetes (T1D), Ulcerative Colitis (UC).

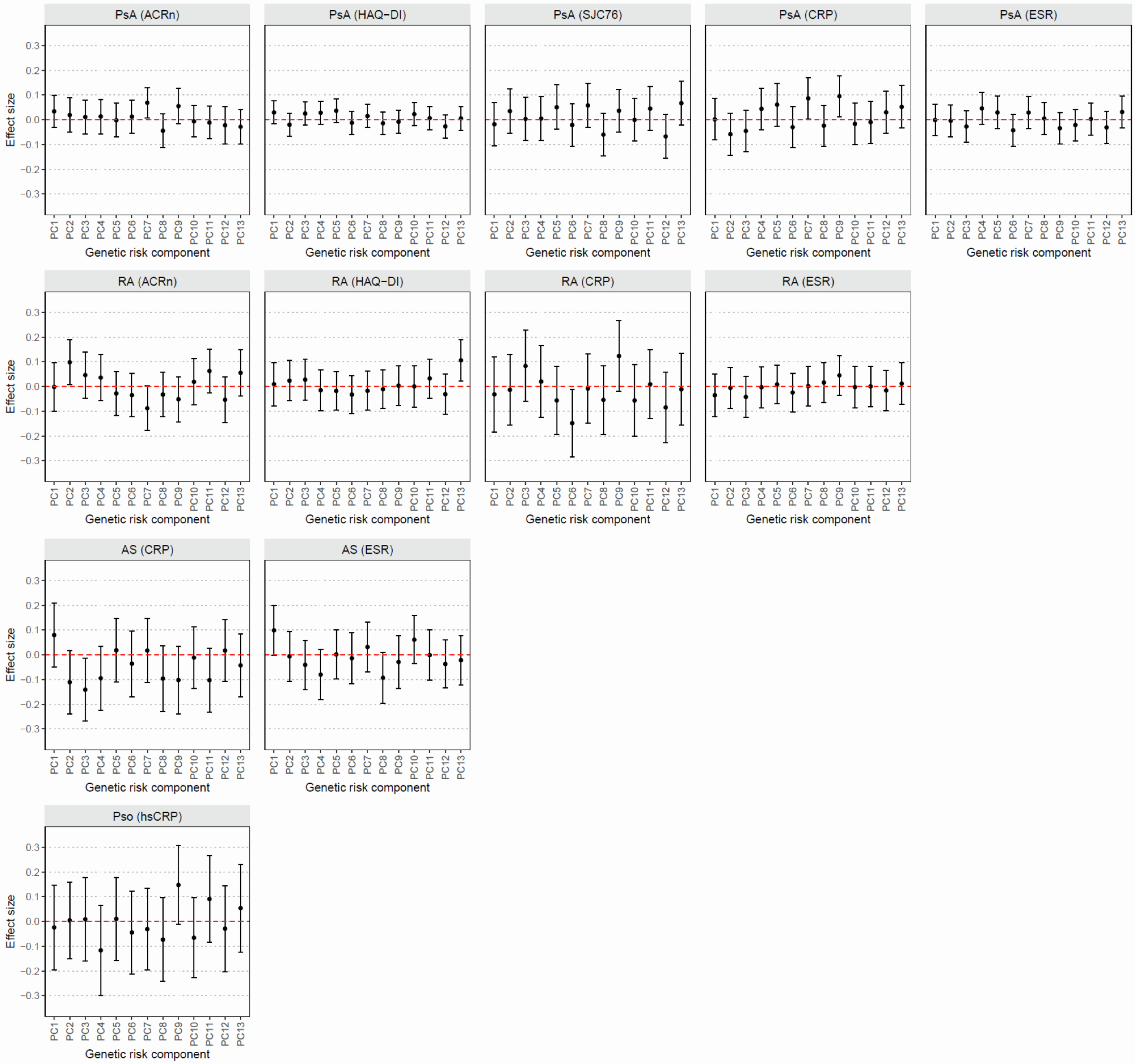

Figure S10: Interaction effect size between treatment and cross-trait polygenic risk scores on secondary outcomes for four indications. Effect sizes are given per standard deviation of the polygenic score. Error bars are 95% confidence intervals. Red dashed line corresponds to effect size = 0.

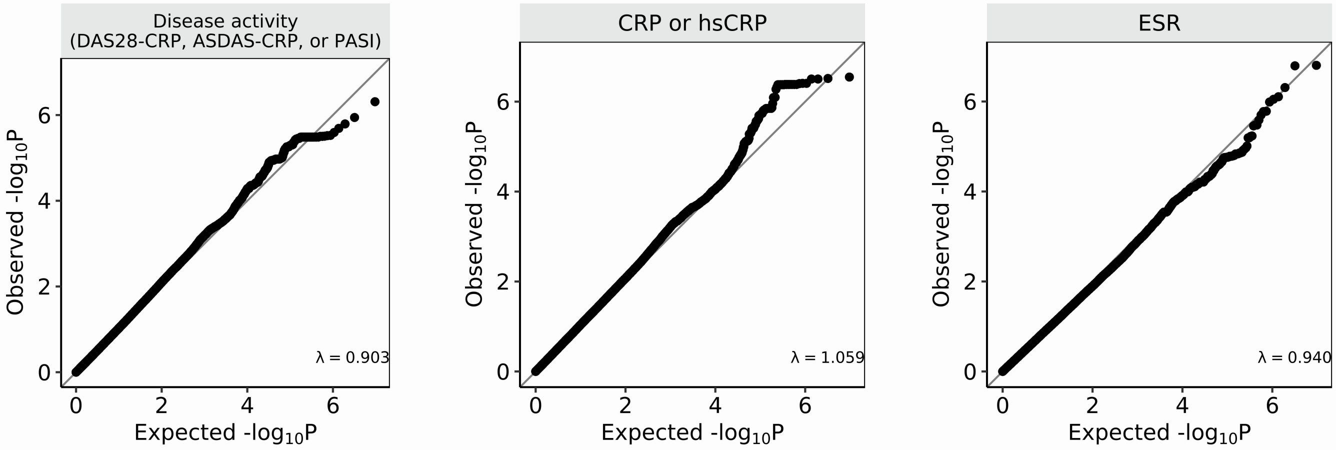

Figure S11: QQ plots for the cross-indication meta-analyses of treatment-by-genotype interaction GWAS for selected primary (disease activity) and secondary (CRP and ESR) outcomes.

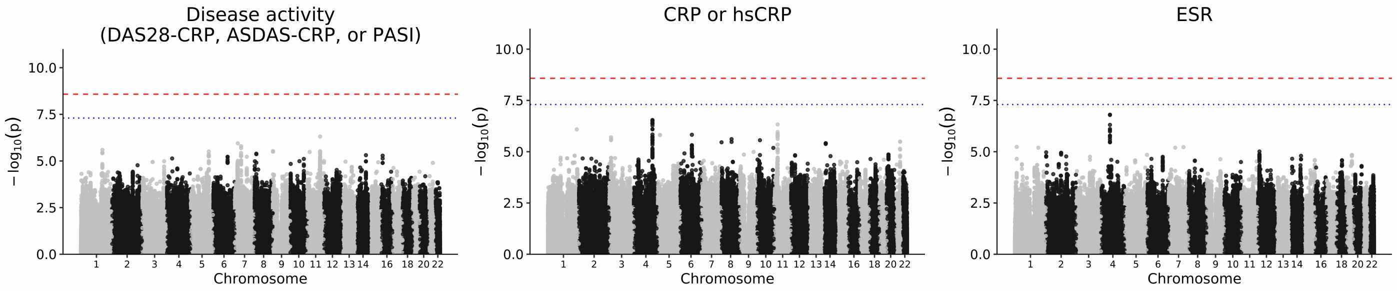

Figure S12: Manhattan plots of cross-indication meta-analyses of treatment-by-genotype interaction GWAS for selected primary (disease activity) and secondary (CRP and ESR) outcomes. The blue line shows genome-wide significance ($p = 5 \times 10^{-8}$) and the red line shows analysis-wide significance controlling for 19 analyses ($p = 2.63 \times 10^{-9}$, i.e. correcting for all individual GWAS and GWAS meta-analyses).

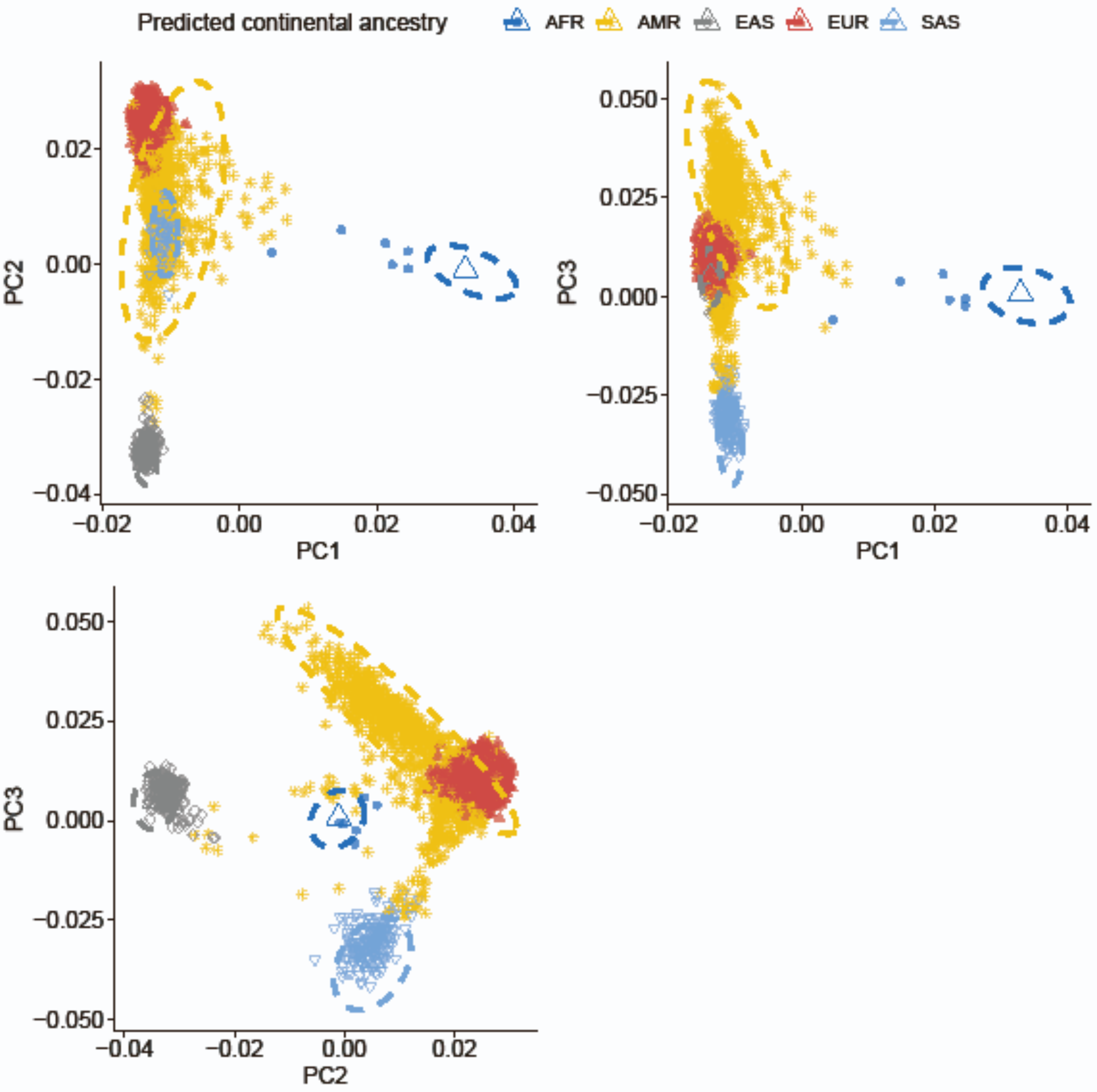

Figure S13: Ancestry assignment by principal component analysis. Each dot represents a patient sample with the color and shape indicating the predicted continental ancestry. The dashed ellipses showed the 95% confidence region of the 1k genome samples in the corresponding ethnic group .

Supplemental Methods

Ethics and consent
This was a post-hoc analysis of existing anonymized clinical trial data collected by Novartis Pharmaceuticals[1]. All trials included in the study are listed in Table S2, and this table includes NCT numbers to allow further information to be found via clinicaltrials.gov.

The site investigators collected the data, Novartis Pharmaceuticals and the University of Oxford conducted the data analyses, and all the authors had access to the data. The studies were approved by the institutional review board or ethics committee at each participating site, and the studies were conducted in accordance with the ethical principles of the Declaration of Helsinki. U.S. sites maintained compliance with Health Insurance Portability and Accountability Act regulations. Eligible patients provided written informed consent.

Power calculations
Power for the largest indication (psoriatic arthritis) was assessed by simulation. Datasets of patients with simulated changes in pre- and post-treatment DAS28-CRP levels were generated under a Gaussian linear model, with number of treatment and placebo cases taken from Table 1, a main treatment effect size of 0.76 units, a genotype-by-treatment interaction term of varying effect size, and a Gaussian error selected to maintain a standard deviation of 0.11 within treatment (all fixed parameters selected to match the previously reported results of study NCT02404350[2]). Power was assessed by the number of simulations that met the primary analysis p-value threshold ($p < 1.25 \times 10$-8) for a given interaction effect size.

Processing and quality control of genotype data
DNA genotyping and base calling were carried out separately for each study on the Illumina Global Screening Array and processed in GenomeStudio. Genotype data for each study underwent a standard quality control pipeline to detect duplicate samples (pi_hat > 0.9), poorly genotyped samples (missingness > 10%), poorly genotyped variants (missingness > 5%), and a pre-defined set of poorly performing probes provided by Illumina. Samples also underwent a sex check to detect mismatches between chromosomal sex and patient reported sex to detect probable sample swaps. Variants were then aligned to the forward strand based on allele complementary and allele frequencies, with ambiguous variants with a minor allele frequency > 20% excluded. Variants were then phased using SHAPEIT[3] (v2.r837) and imputed using IMPUTE2[4] (v2.3.2) with 1000 Genomes haplotypes used as the reference (Phase 3 b37[5]).

After imputation, we carried out another level of QC on each study. Variants with an info score of less than 0.7 or a minor allele frequency of less than 1% were removed. Finally, hard calls were generated by setting genotypes to their highest posterior value, and setting calls where the best guess posterior was less than 90% to missing. All samples with > 10 % missing data at SNPs with < 10% missing data within each study were removed from further analyses, as well as SNPs with a Hardy-Weinberg Equilibrium (HWE)-threshold of 1×10^{-6} or a minor allele frequency of less than 0.25 %. Related and duplicated samples (pi_hat > 0.125) were also filtered out. The individual studies for each indication were then merged into a

single file, and triallelic SNPs were removed, as were variants with a minor allele frequency <1% across an entire indication.

Studies were merged by indication and PCA ancestry (see the next section for a description of how PCA ancestry was assigned) group, and a final round of QC was performed within these groups. All variants with missing genotype rate > 2%, failing the HWE p-value-threshold of 1×10^{-6}, having a minor allele frequency of less than 1 %, or with significantly different allele frequencies across the batches based on a false-discovery rate (FDR) threshold of 1×10^{-5} for each test were removed. In addition, individuals with missing genotype rate >2 %, with inconsistent reported and genetically determined sex, or that had a significantly higher or lower inbreeding coefficient (F) (calculated using the '--het' option in plink) at FDR 0.05, showed a relatedness with pi_hat > 0.18, were removed.

PCA analysis and PCA ancestry assignment

Merged samples were classified into continental ancestry groups (which we refer to as "PCA ancestry") using principal component analysis (PCA) using a 1000 Genome reference set. More specifically, we carried out principal component analysis on the 1000 Genome phase 3 samples, using only those variants that were present in both 1000 Genome and the GSA chip. Each of our study samples was then projected onto the first two principal component axes, and for each sample we calculated a z-scores for each of the five 1000 Genomes continental groups (EUR/Europeans, AFR/Africans, SAS/South Asians, EAS/East Asians, AMR/Admixed Americas), based on the distance of the sample to the centre of the continental groups. Each sample was assigned to one of these five PCA ancestry groups according to the minimal z-score. The position of our samples in this PCA space, coloured by ancestry alignment, is shown in Supplementary Figure S13. PCA within each assigned ancestry group samples were also performed and the first three principal components were used to control for population stratification in the following analysis.

Imputation of common HLA alleles

Common HLA alleles were imputed to 4-digit resolution using the HIBAG package[6] (v3.12), using a prediction model built for the Global Screening Array. We imputed alleles separately within each PCA ancestry cluster, using the European model to predict EUR samples, the Asian model to predict SAS and EAS samples, the African model to predict AFR samples and the Hispanic model to predict AMR samples. Only calls with posterior probability >0.5 were included (otherwise the call was set to missing), and the maximum posterior allele assignment as used. HLA alleles were filtered to include only those with an allele frequency greater than 1% within each ancestry cluster, and a new variable for the dosage of each HLA allele were generated for downstream testing.

Association analysis and follow-up

A GWAS was run using plink (v1.9b) for each primary and secondary outcome within the four indication and across each PCA ancestry with at least 100 samples for that indication. The primary outcomes were disease activity scores (DAS28-CRP for RA and PsA, ASDAS-CRP for AS, PASI for Pso), and secondary outcomes included biomarkers (CRP, ESR for RA, PsA and AS as well as hsCRP for Pso), a drug response measure (ACRn, for RA and PsA), a measure of disability (HAQ-DI for RA and PsA) and a measure of swollen joint count of 76

joints (SJC76 for PsA, see table below for full description). For each endpoint, the GWAS was carried out separately within each PCA ancestry group with at least 100 samples, and then a combined result for that endpoint was generated by meta-analysing the results across PCA ancestry groups using a fixed effect inverse-variance weighted approach (with effect size estimates and standard errors as input). The sample-size cut-off of 100 as chosen to prevent false positives due to underestimated standard errors, which are common with smaller sample sizes [7]. Three outcomes (CRP, ESR and disease activity scores) were also meta-analyzed across indications using a sample-size-weighted approach (with p-value and direction of effect as input, weighted by sample size) to account for the possibility of differences in effect size across indications. All meta-analyses were calculated using the program METAL[8]. Prior to analysis, PASI scores were transformed to normal quantiles, biomarkers (CRP, hsCRP and ESR) were both log(x+1) transformed, and ACRn was transformed using log[(100 - x) + 1]. A summary of the outcomes tested, with descriptions, ranges and transformations is below:

**Indication(s)**	**Outcome**	**Description**	**Primary/secondary**	**Range**	**Transformation**
PsA/RA	DAS28-CRP	Disease Activity Score-28 with CRP	Primary	0 – X*	None
PsA/RA	ACRn	% improvement in American College of Rheumatology multidimensional response score	Secondary	0 - 100	log[(100 - x) + 1]
PsA/RA/AS/Pso	CRP/hsCRP	Serum C-reactive protein measurement	Secondary	0 – X*	log(x + 1)
PsA/RA/AS	ESR	Blood erythrocyte sedimentation rate	Secondary	0 – X*	log(x + 1)
PsA/RA	HAQ-DI	Health Assessment Questionnaire Disability Index	Secondary	0 - 3	None
PsA	SJC76	Swollen Joint Count 76	Secondary	0 - 76	None
AS	ASDAS-CRP	Ankylosing Spondylitis Disease Activity Score with CRP	Primary	0 - X*	None
Pso	PASI	Psoriasis Area and Severity Index	Primary	0 - 72	Quantile normalized

*biomarker values are theoretically unbounded, values here are maximum in our data

Each GWAS assessed the effects of gene-treatment interaction on each outcome of interest at 16 weeks for PsA, RA and AS and at 12 weeks for Pso (i.e. the primary readout time-points in the original study protocols) using linear regression, adjusting for baseline measure (where available), demographics (age, sex and BMI categorized into four groups according

to the WHO classification[9]), the top 3 genetic PCs (selected based on the scree plots within each ethnicity), study population, use of methotrexate (MTX) and tumor necrosis factor inhibitor (TNFi) medications (where available), as well as SNP and treatment main effects. "Treatment" was defined as any patient randomized to reive secukinumab, regardless of dosing regimen. Subjects with outcome measures within 2 weeks of the target times were included in the analysis. All models were adjusted for baseline except ACRn, as it was used in the calculation of the outcome. The model fit for analysis was:

outcome_endpoint = outcome_baseline + age + sex + BMI + study + MTX + TNFi + PC1 + PC2 + PC3 + treatment + SNP + treatment × SNP

In addition, for all analyses we also recorded the summary statistics for the main effect (I.e. the coefficient for SNP in the above model), and the joint effect p-value (a hypothesis testing comparing whether either of the SNP or treatment × SNP effects are non-zero, as described in [10]).

Initially, all analyses were carried out in Plink using standard ordinary least squares (OLS) linear regression. However, it is known that model misspecification, and in particular heteroscedasticity (i.e. a violation of the homogeneity of variance assumption) can lead to false negatives and false positives in genetic interaction models [11, 12]. We tested all combinations of indication, outcome and ancestry group for heteroscedasticity under the null using the Breusch-Pagan test [13] and found significant deviation from homogeneity of variance in many of our models (Supplementary Table S11). We inspected -log10 QQ plots for all indication and outcomes (Supplementary Figures S2, S3 and S11), and saw evidence of significant inflation or deflation for three analyses (deflation in PsA ACRn and Pso PASI, and inflation for RA DAS28-CRP). We re-ran the full genome-wide association analyses for these three traits using the same model but with standard errors and p-value estimated using a heteroscedasticity robust HC4 sandwich estimator[14], implemented in the R package sandwich. This produced well-calibrated QQ plots for PsA ACRn and Pso PASI (black dots in Supplementary Figure S2 and S3). However, the QQ plot for RA DAS28CRP remained inflated even in the robust analysis. We carried out a permutation analysis to test if this inflation was caused by miscalibrated p-values under the null, by permuting the genotypes for the genome-wide-significant variants 100,000 times and refitting the robust regression test to generate an empirical null distribution. This analysis showed that the genome-wide significant associations in this analysis were false positive artefacts (having empirical permuted $p > 1e-5$). These false positives were driven by extremely significant results in a single ancestry group, so to remove them we filtered out results that had inconsistent associations across the ancestry groups by applying a filter for heterogeneity of effect ($p < 0.05$) using Cochran's Q[15]. This filtered dataset produced a well-calibrated QQ plot and removed the false positive artefacts, though we should note a limitation of this approach is that it may also remove truly population-specific associations as well. Our final set of summary statistics thus consisted of robust regression results for Pso PASI and PsA ACRn, robust regression results with heterogeneity of effect filtering for RA DAS28CRP, and OLS results for all other analyses. The final regression test selected for each outcome is shown in

Supplementary Table S3. We also tested all genome-wide significant associations from any analysis in both OLS and robust regression as a post-hoc sensitivity check.

We calculated the proportion of variance explained by treatment-gene interactions (the GxE heritability) using LDSC[16] with default parameters, as detailed by Shin *et al*[17].

Testing of polygenic risk scores for susceptibility

To produce a set of associations to immune or inflammatory diseases, for the purposes of calculating polygenic risk scores (PRS), 12 studies of immune mediated diseases with full summary statistics were identified from the EBI GWAS catalogue[18]. Information on studies included are in Supplementary Table S7. Summary statistics were imputed using the ssimp package[19] using ancestry-matched 1000 Genomes reference sets. We calculated polygenic risk scores using the genome-wide Bayesian shrinkage model PRS-CS[20], using the settings suggested in the tutorial (including setting the shrinkage parameter phi=1e-2, as suggested for smaller GWAS). The 12 scores were then calculated for each sample in our study using plink (v1.9b). We also calculated polygenic risk scores for the 1000 Genomes samples, and validated that the correct scores were associated with the correct indications (e.g. the PsA score was higher in the PsA indication than in the 1000 Genomes samples for the same PCA ancestry, data not shown).

Decomposed polygenic risk scores were calculated using betas for each of the 13 principal component basis of shared genetic risk for immune-mediated diseases identified by Burren *et al*[21]. We attempted to regenerate principal components for our 12 IMD studies using the same cupcake method, but this did not produce a sparse basis, likely due to the low sample sizes (Burren *et al* recommends N > 6000, which not all of our 12 studies met) and the fact that some of our studies were not genome-wide (as we included studies with targeted Immunochip data). We thus used the basis generated by Burren *et al*, as at least one of the 13 Burren *et al* components was associated with each of our four indications, suggesting that the genetic components identified covered the relevant region of genetic risk for our samples. We calculated polygenic scores for each sample in each of our four indications, using the approach described in Burren *et al*.

We assessed the effects of PRS-treatment interaction, as well as main and joint effects, on each outcome of interest for the four indications using linear model, adjusting for the same covariates in the GWAS model, including baseline measures, demographics (age, sex and WHO categories of BMI), Study ID, the top 3 genetic PCs, use of MTX, and TNFi medications (where available). The polygenic scores were normalized to zero-mean and unit-variance for comparison purpose. We used robust sandwich estimators for Pso PASI, RA DAS-28-CRP and PsA ACRn, to mirror the approach used for the GWAS analysis. The association model was run for each outcome within the four indication and across each ethnicity (European, Admixed American and East Asian) in populations with at least 100 samples. The results from the individual models were then meta-analyzed across ethnicity using a fixed-effect inverse-variance-weighted standard error approach. We carried out multiple testing correction using the Benjamini-Hochberg procedure to preserve a false discovery rate of 0.05.

Association testing of HLA alleles

The same approach to testing PRS-treatment interactions was also used to test HLA-treatment interactions.

Supplemental References:

1. Mallon, A.-M., Häring, D.A., Dahlke, F., Aarden, P., Afyouni, S., Delbarre, D., El Emam, K., Ganjgahi, H., Gardiner, S., Kwok, C.H., et al. (2021). Advancing data science in drug development through an innovative computational framework for data sharing and statistical analysis. BMC Med. Res. Methodol. *21*, 250.

2. Mease, P., van der Heijde, D., Landewé, R., Mpofu, S., Rahman, P., Tahir, H., Singhal, A., Boettcher, E., Navarra, S., Meiser, K., et al. (2018). Secukinumab improves active psoriatic arthritis symptoms and inhibits radiographic progression: primary results from the randomised, double-blind, phase III FUTURE 5 study. Ann. Rheum. Dis. *77*, 890–897.

3. Delaneau, O., Coulonges, C., and Zagury, J.-F. (2008). Shape-IT: new rapid and accurate algorithm for haplotype inference. BMC Bioinformatics *9*, 540.

4. Howie, B.N., Donnelly, P., and Marchini, J. (2009). A flexible and accurate genotype imputation method for the next generation of genome-wide association studies. PLoS Genet. *5*, e1000529.

5. 1000 Genomes Project Consortium, Auton, A., Brooks, L.D., Durbin, R.M., Garrison, E.P., Kang, H.M., Korbel, J.O., Marchini, J.L., McCarthy, S., McVean, G.A., et al. (2015). A global reference for human genetic variation. Nature *526*, 68–74.

6. Zheng, X., Shen, J., Cox, C., Wakefield, J.C., Ehm, M.G., Nelson, M.R., and Weir, B.S. (2014). HIBAG--HLA genotype imputation with attribute bagging. Pharmacogenomics J. *14*, 192–200.

7. Lin, L. (2018). Bias caused by sampling error in meta-analysis with small sample sizes. PLoS One *13*, e0204056.

8. Willer, C.J., Li, Y., and Abecasis, G.R. (2010). METAL: fast and efficient meta-analysis of genomewide association scans. Bioinformatics *26*, 2190–2191.

9. World Health Organization (2000). Obesity: Preventing and Managing the Global Epidemic : Report of a WHO Consultation (World Health Organization).

10. Kraft, P., Yen, Y.-C., Stram, D.O., Morrison, J., and Gauderman, W.J. (2007). Exploiting gene-environment interaction to detect genetic associations. Hum. Hered. *63*, 111–119.

11. Rao, T.J., and Province, M.A. (2016). A framework for interpreting type I error rates from a product-term model of interaction applied to quantitative traits. Genet. Epidemiol. *40*, 144–153.

12. Voorman, A., Lumley, T., McKnight, B., and Rice, K. (2011). Behavior of QQ-plots and genomic control in studies of gene-environment interaction. PLoS One *6*, e19416.

13. Breusch, T.S., and Pagan, A.R. (1979). A Simple Test for Heteroscedasticity and Random Coefficient Variation. Econometrica *47*, 1287–1294.

14. Cribari-Neto, F. (2004). Asymptotic inference under heteroskedasticity of unknown form. Comput. Stat. Data Anal. *45*, 215–233.

15. Cochran, W.G. (1954). The Combination of Estimates from Different Experiments. Biometrics *10*, 101–129.

16. Bulik-Sullivan, B.K., Loh, P.-R., Finucane, H.K., Ripke, S., Yang, J., Schizophrenia Working Group of the Psychiatric Genomics Consortium, Patterson, N., Daly, M.J., Price, A.L., and Neale, B.M. (2015). LD Score regression distinguishes confounding from polygenicity in genome-wide association studies. Nat. Genet. *47*, 291–295.

17. Shin, J., and Lee, S.H. (2021). GxEsum: a novel approach to estimate the phenotypic variance explained by genome-wide GxE interaction based on GWAS summary statistics for biobank-scale data. Genome Biol. *22*, 183.

18. Buniello, A., MacArthur, J.A.L., Cerezo, M., Harris, L.W., Hayhurst, J., Malangone, C., McMahon, A., Morales, J., Mountjoy, E., Sollis, E., et al. (2019). The NHGRI-EBI GWAS Catalog of published genome-wide association studies, targeted arrays and summary statistics 2019. Nucleic Acids Res. *47*, D1005–D1012.

19. Rüeger, S., McDaid, A., and Kutalik, Z. (2018). Evaluation and application of summary statistic imputation to discover new height-associated loci. PLoS Genet. *14*, e1007371.

20. Ge, T., Chen, C.-Y., Ni, Y., Feng, Y.-C.A., and Smoller, J.W. (2019). Polygenic prediction via Bayesian regression and continuous shrinkage priors. Nat. Commun. *10*, 1776.

21. Burren, O.S., Reales, G., Wong, L., Bowes, J., Lee, J.C., Barton, A., Lyons, P.A., Smith, K.G.C., Thomson, W., Kirk, P.D.W., et al. (2020). Genetic feature engineering enables characterisation of shared risk factors in immune-mediated diseases. Genome Med. *12*, 106.
