# Supplementary material for: Response to anti-IL17 therapy in inflammatory disease is not strongly impacted by genetic background
Source: Am J Hum Genet. 2023 Sep 1;110(10):1817–24. doi: 10.1016/j.ajhg.2023.08.010 (PMC10577077; doi:10.1016/j.ajhg.2023.08.010)
Supplement: Document S2. Article plus supplemental information [file mmc3.pdf]

Response to anti-IL17 therapy in inflammatory disease is not strongly impacted by genetic background

Authors

Cong Zhang, Konstantin Shestopaloff, Benjamin Hollis, ..., Enrico Ferrero, Marc Vandemeulebroecke, Luke Jostins-Dean

Correspondence

marc.vandemeulebroecke@novartis.com (M.V.), luke.jostins@kennedy.ox.ac.uk (L.J.-D.)

We investigated the role of genetics in clinical response to secukinumab in 5,218 individuals from 19 clinical trials across four indications. We found no association between response and common variants, imputed HLA alleles, polygenic disease susceptibility risk, or cross-disease shared genetic risk. Anti-IL17 therapy appears equally effective regardless of genetics.

Zhang et al., 2023, The American Journal of Human Genetics *110*, 1817–1824
October 5, 2023

© 2023 The Authors.

https://doi.org/10.1016/j.ajhg.2023.08.010

CellPress

Response to anti-IL17 therapy in inflammatory disease is not strongly impacted by genetic background

Cong Zhang,[1,14] Konstantin Shestopaloff,[2,3,14] Benjamin Hollis,[4] Chun Hei Kwok,[5] Claudia Hon,[6] Nicole Hartmann,[7] Chengeng Tian,[1] Magdalena Wozniak,[8] Luis Santos,[9] Dominique West,[10] Stephen Gardiner,[2] Ann-Marie Mallon,[9] Aimee Readie,[11] Ruvie Martin,[11] Thomas Nichols,[2] Michael T. Beste,[6] Jonas Zierer,[12] Enrico Ferrero,[12,15] Marc Vandemeulebroecke,[13,15,*] and Luke Jostins-Dean[2,4,15,*]

Summary

Response to the anti-IL17 monoclonal antibody secukinumab is heterogeneous, and not all participants respond to treatment. Understanding whether this heterogeneity is driven by genetic variation is a key aim of pharmacogenetics and could influence precision medicine approaches in inflammatory diseases. Using changes in disease activity scores across 5,218 genotyped individuals from 19 clinical trials across four indications (psoriatic arthritis, psoriasis, ankylosing spondylitis, and rheumatoid arthritis), we tested whether genetics predicted response to secukinumab. We did not find any evidence of association between treatment response and common variants, imputed HLA alleles, polygenic risk scores of disease susceptibility, or cross-disease components of shared genetic risk. This suggests that anti-IL17 therapy is equally effective regardless of an individual's genetic background, a finding that has important implications for future genetic studies of biological therapy response in inflammatory diseases.

Response to biological therapy in inflammatory disease is typically heterogeneous, and even in highly successful and widely used treatments (such as tumor necrosis factor inhibitor [TNFi] therapies), a significant fraction of participants fail to respond to treatment.[1] A common hypothesis is that heterogeneity in response reflects genetic differences between individuals or, relatedly, genetically distinct disease subtypes with different molecular etiologies.[2] If this hypothesis is true and genetic biomarkers for drug response can be identified, this could lead to new understanding of the biology of drug response or discovery of biomarkers to stratify subgroups of participants to specific treatments to increase response rates.[3–5]

A further hypothesis is that genetic pathways that place individuals at risk of disease may also influence their response to therapy, and this may be captured by polygenic scores of disease susceptibility.[6,7] This is even more plausible where the pathway being treated is known to play an important role in disease susceptibility, as for anti-interleukin-17 (IL17) and anti-IL23 therapies.[8,9] When multiple indications are treated by the same drug, the same risk variants may have differing directions of effect for related diseases.[10] To overcome this pleiotropy of inflammatory pathways, genetic risk can be modeled across multiple inflammatory diseases using several orthogonal genetic components[11] that may correlate with treatment response.

Multiple genome-wide association studies (GWASs) of response to biological therapies have been published.[12–15] These primarily consist of post-approval studies of drugs in regular use with most studying TNFi therapies. The most consistent association known is between HLA-DQA1*05 alleles and development of anti-drug antibodies.[14–16] However, relatively low sample sizes, small number of associations, and lack of consistent cross-replication of effects between these studies has made it difficult to draw firm conclusions of the role of genetics in treatment response. In addition, these studies included only participants under active treatment and thus could not discriminate between prognostic biomarkers (which correlate with outcomes independently of treatment) and predictive biomarkers (which correlate with response to a specific treatment).[17] In this study, we used data from clinical trial participants, randomized to secukinumab or placebo, to test for predictive biomarkers.

Secukinumab (brand name Cosentyx) is a widely used therapy for treating inflammatory diseases by blocking the pro-inflammatory IL-17 signaling pathway. It has been approved for use in plaque psoriasis (Pso), ankylosing spondylitis (AS), psoriatic arthritis (PsA), and other

[1]China Novartis Institutes for Bio-medical Research CO., Shanghai, China; [2]Big Data Institute, Li Ka Shing Centre for Health Information and Discovery, Nuffield Department of Medicine, University of Oxford, Oxford, UK; [3]Department of Statistics, University of Oxford, Oxford, UK; [4]Kennedy Institute of Rheumatology, University of Oxford, Oxford, UK; [5]Nuffield Department of Population Health, University of Oxford, Oxford, UK; [6]Novartis Institutes for BioMedical Research, 220 Massachusetts Avenue, Cambridge, MA 02139, USA; [7]Novartis Pharma AG, Lichtstrasse 35, Basel, CH, Switzerland; [8]Novartis Ireland Limited, Dublin, Ireland; [9]The Alan Turing Institute, London, UK; [10]Radcliffe Department of Medicine, University of Oxford, Oxford, UK; [11]Novartis Pharmaceuticals Corporation, East Hanover, NJ, USA; [12]Novartis Institutes for BioMedical Research, Basel, CH, Switzerland; [13]Novartis Pharma AG, Global Drug Development, Basel, CH, Switzerland
[14]These authors contributed equally
[15]These authors contributed equally
*Correspondence: marc.vandemeulebroecke@novartis.com (M.V.), luke.jostins@kennedy.ox.ac.uk (L.J.-D.)
https://doi.org/10.1016/j.ajhg.2023.08.010.
© 2023 The Authors. This is an open access article under the CC BY license (http://creativecommons.org/licenses/by/4.0/).

inflammatory conditions and has been extensively studied in clinical trials (including in indications for which it has not been approved, such as rheumatoid arthritis [RA]). Individual participants' responses to treatment are heterogeneous within indications, with response rates of 81.6% for Pso,[18] 62.6% for PsA,[19] 60.5% for AS,[20] and 30.7% for RA.[21] Even for the same disease and outcome, there can be substantial variability in response rates from study to study in real-world settings,[22] and there is some evidence that response rates can vary between countries, sexes, and disease subtypes.[23,24]

There have, to date, been no genome-wide studies of response to anti-IL17 therapy, though two small studies in psoriasis have investigated candidate genes, primarily within the human leukocyte antigen (HLA) locus.[23,25,26] The clinically significant but heterogeneous response to a biologic therapy, measured in many clinical trials, provides a unique dataset to test for predictive biomarkers. Here, we tested whether individual genetic variants (including SNPs and HLA alleles), polygenic risk scores (PRSs) of susceptibility, or components of genetic risk are associated with response to anti-IL17 therapy in a large dataset from multiple randomized placebo-controlled trials across four diseases.

We gathered genetic and clinical data from 19 clinical trials of anti-IL17 therapy with secukinumab (Table S1) across four indications (PsA, RA, AS, and Pso). We used continuous measures of disease activity as our primary outcomes: the disease activity score 28 with C-reactive protein (DAS28-CRP) for PsA and RA,[27] the AS disease activity score with CRP (ASDAS-CRP) for AS,[28] and the psoriasis area and severity index (PASI) score for Pso.[29] After quality control, we had complete clinical and genotype data on 5,218 participants, including 4,063 treated with anti-IL17 therapy and 1,151 placebo controls. Demographic and clinical characteristics of these participants are shown in Table 1 and are broken down by treatment, randomization status, and exclusion status in Table S2.

Power calculations showed that these samples sizes provide enough power to detect common variants or risk scores that exert a clinically meaningful effect. For the best-powered indication (PsA, $n = 2,006$), our primary analysis had >80% power to detect a common variant that increases the difference between DAS28-CRP in treatment vs. controls by 0.06 units per allele (Figure S1), equivalent to an 8% modification in the effect of treatment.

For our primary analyses, we carried out a genome-wide treatment-by-genotype interaction study for each of the four indications separately. We stratified participants by their predicted continental ancestry (PC ancestry, defined based on their distance from nearest continental population on a principal component analysis [PCA] of samples from the 1000 Genomes Project[30]) and meta-analyzed the results across ancestry groups (after removing ancestry groups with low sample size). We included three genetic principal components as well as known demographics and other confounders in the regression model (see supplemental methods for details). We detected evidence of miscalibration in test statistics in the quantile-quantile (Q-Q) plots for two of these analyses (Pso PASI and RA DAS28-CRP, Figure S2) due to model misspecification driven by heteroscedasticity, a known problem with gene-treatment interaction models.[31,32] We thus replaced these two analyses with robust regression analyses[33] (see supplemental methods for details).

Linkage disequilibrium score (LDSC[34,35]) regression did not show a significant treatment-by-genome heritability term, though confidence intervals were large (Table S3). No variant passed study-wide significance correcting for four tests ($p < 1.25 \times 10^{-8}$) (Figure 1). We also tested variants previously associated with response to other biologic therapies and did not find any significant variant-treatment interactions after correcting for multiple testing (Table S4). One variant, rs11762062, met genome-wide but not study-wide significance for DAS28-CRP in RA ($p = 2.08 \times 10^{-8}$, Table S5), but this variant did not replicate in the PsA DAS28-CRP analysis ($p = 0.332$).

To maximize power, we also carried out a cross-indication meta-analysis of these results. We meta-analyzed the primary outcomes using a sample-size weighted Z score meta-analysis in order to make less strict assumptions about comparability of the scale of effects across indications. We did not find any study-wide or genome-wide significant associations (Figure 2). The sample sizes in this case were large enough to make inferences using the LDSC heritability analysis Table S3), which showed that the variability in disease activity explained by genotype-treatment interactions, when averaged across indications, must be relatively small (upper bound of 28%).

Next, we tested for interactions between drug response and imputed HLA alleles. We did not find any significant associations after adjusting for the 489 allele/outcome combinations tested (Figure 3; Table S6) and did not find any nominally significant associations with any of the major risk HLA alleles for the four indications, with the HLA-Cw6 (C*06) allele previously associated with response to anti-IL17 therapy[26] or with the HLA-DQA1*05 alleles previously associated with TNFi immunogenicity[14–16] (Table S4).

To test the hypothesis that disease-susceptibility variants could influence drug response, we also calculated PRSs for 11 inflammatory diseases, including the four indications and seven other related diseases (celiac disease, Crohn disease, multiple sclerosis, primary biliary cirrhosis, systemic lupus erythematosus, type 1 diabetes, and ulcerative colitis; see Table S7 for references). In addition, we calculated risk scores based on shared orthogonal genetic components of immune and inflammatory disease risk following Burren et al.[11] However, none of these risk scores predicted response to anti-IL17 therapy (Figure 4; Tables S8 and S9).

Our primary analysis used clinical disease activity scores, which are, in most cases, composites of multiple measures, including numerical ratings of clinician- or patient-assessed disease state, objective measures of disease symptoms (such as swollen joint counts), and biomarker measures. We also

Table 1. Clinical and demographic statistics of participants included in this study, broken down by indication; treated cases include any participants randomized to receive secukinumab, regardless of dosing regimen

	AS (n = 754)	PsA (n = 2,006)	Pso (n = 1,636)	RA (n = 822)
Primary outcome	ASDAS-CRP	DAS28-CRP	PASI	DAS28-CRP
Baseline value of primary outcome (median [min, max])	3.63 [0.528, 6.22]	4.59 [1.37, 8.28]	4.59 [1.37, 8.28]	5.72 [2.58, 8.01]
Delta in primary outcome (median [min, max])	−0.866 [−4.62, 1.84]	−1.05 [−5.25, 2.49]	−1.05 [−5.25, 2.49]	−1.22 [−5.58, 3.03]
# of studies	4	6	4	5
# of treated cases	506	1,483	1,495	583
# of placebo cases	248	523	141	239
Age (median [min, max])	42.0 [17.0, 82.0]	50.0 [16.0, 83.0]	45.0 [15.0, 87.0]	55.0 [19.0, 84.0]
Sex				
Female	249 (33.0%)	1,029 (51.3%)	507 (31.0%)	661 (80.4%)
BMI category				
Underweight	16 (2.1%)	9 (0.4%)	15 (0.9%)	33 (4.0%)
Normal weight	256 (34.0%)	459 (22.9%)	374 (22.9%)	308 (37.5%)
Overweight	213 (28.2%)	681 (33.9%)	576 (35.2%)	239 (29.1%)
Obese	269 (35.7%)	857 (42.7%)	671 (41.0%)	242 (29.4%)
Methotrexate use at recruitment				
Yes	87 (11.5%)	876 (43.7%)	not used	741 (90.1%)
Previous anti-TNF treatment failure at recruitment				
Yes	530 (70.3%)	1,517 (75.6%)	not used	not used
PC ancestry				
AMR	117 (15.5%)	200 (10.0%)	229 (14.0%)	193 (23.5%)
EAS	<5 (<0.6%)	17 (0.8%)	77 (4.7%)	110 (13.4%)
EUR	632 (83.8%)	1,741 (86.8%)	1,234 (75.4%)	499 (60.7%)
SAS	<5 (<0.6%)	48 (2.4%)	96 (5.9%)	17 (2.1%)
AFR	<5 (<0.6%)	–	–	<5 (<0.4%)

Delta is the change in primary outcome between baseline and assessment week (week 12 for Pso, week 16 for all other indications). AS, ankylosing spondylitis; PsA, psoriatic arthritis; Pso, psoriasis; RA, rheumatoid arthritis; BMI, body mass index; PC ancestry, ancestry assigned by principal component analysis; AMR, admixed American; EAS, east Asian; EUR, European; SAS, south Asian; AFR, African.

ran 12 further genome-wide treatment-by-genotype interaction analyses for a range of secondary outcomes, including laboratory-based and self-reported measures (see supplemental methods for a full list and Figures S3–S7 for results; note that one outcome, PsA ACRn (percentage improvement in American College of Rheumatology multidimensional response score), was replaced with a robust regression analysis). We did not see any study-wide significant associated variants correcting for 16 (4 primary and 12 secondary) analyses ($p < 3.12 \times 10^{-9}$), and none of the disease/endpoint pairs showed a Bonferroni-corrected significant treatment-by-genome heritability component in the LDSC analysis (Table S3). We also carried out HLA and PRS analyses with secondary outcomes, none of which showed significant associations (Figures S8–S10). We meta-analyzed CRP and erythrocyte sedimentation rate (ESR) across indications and did not find any study-wide significant associations (Figures S11 and S12) or appreciable heritability (Table S3).

The aim of this paper was to test for predictive, not prognostic, effects of genetics on drug response. However, to maximize power, we also carried out a joint predictive-prognostic analysis using a joint test[36] (i.e., a combined test for either a main effect of genotype on outcome regardless of treatment status and an interaction effect with treatment). This did not produce any study-wide significant results, and three genome-wide but not study-wide significant results are presented in Table S10. We did find that a previously reported SNP (rs7195994) association with prognostic response to TNFi at *FTO*[13] replicated at nominal significance ($p < 0.05$) in our prognostic main effect analysis (Table S4).

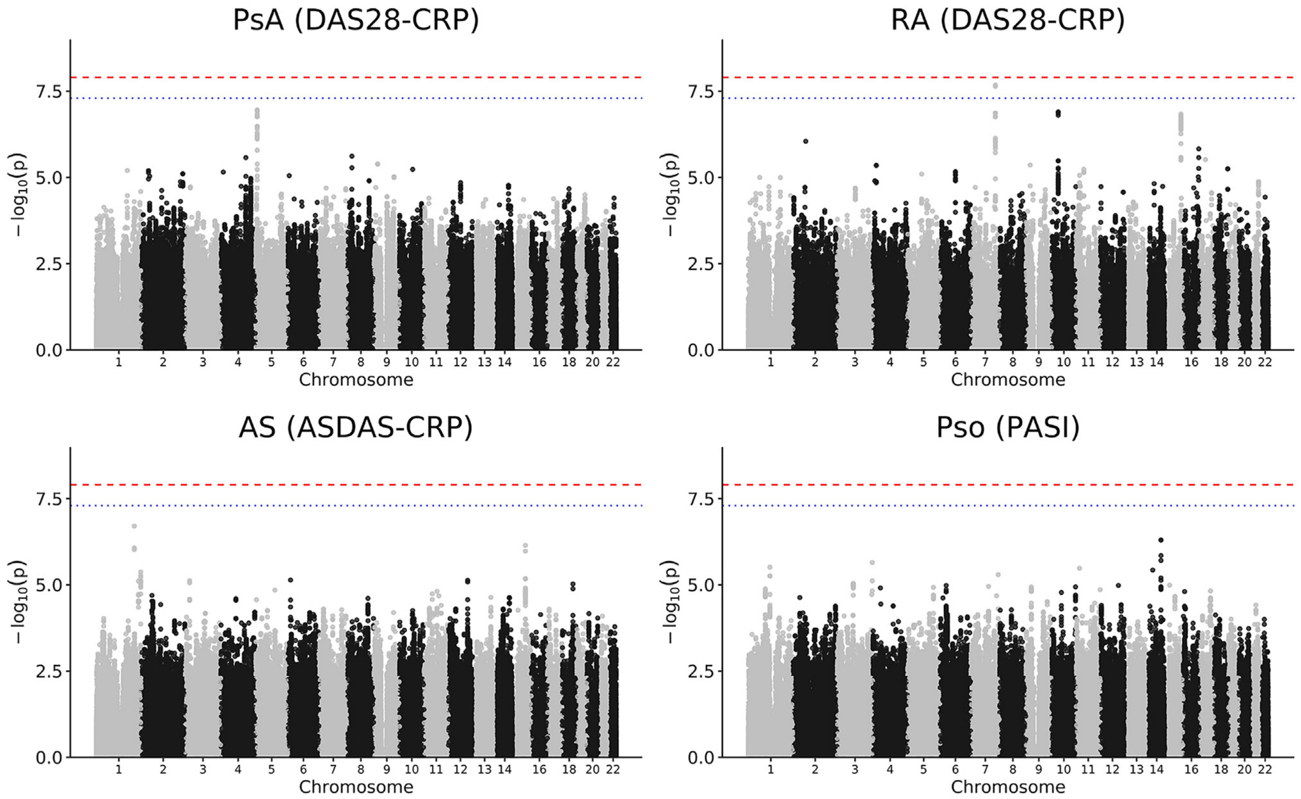

Figure 1. Manhattan plots of treatment-by-genotype interaction GWASs for the primary outcome of each of the four indications tested
p values are from linear regression. The blue line shows genome-wide significance ($p = 5 \times 10^{-8}$) and the red line shows analysis-wide significance controlling for four indications ($p = 1.25 \times 10^{-8}$). Each plot shows a meta-analysis of all ethnicities with a sample size of at least 100.

Based on our findings, we can rule out several important hypotheses about the genetics of response to anti-IL17 therapy. We have shown that common variants do not have a moderate-to-large effect in response to anti-IL17 therapy, which includes common SNPs and also, more surprisingly, common HLA alleles. We can also exclude a large effect on response from the genetic pathways that influence susceptibility, either measured by PRSs for susceptibility to individual inflammatory diseases or by scores that reflect shared genetic pathways across inflammatory diseases. This adds strong evidence to support the claim that genetic heterogeneity is not a large driver of response to anti-IL17 therapy and that secukinumab is equally effective regardless of genetic background.

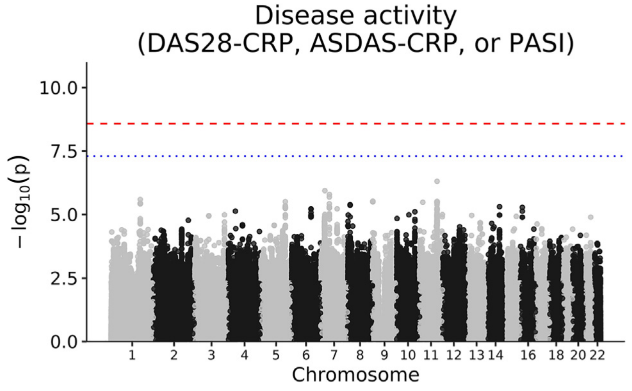

Figure 2. Manhattan plots of cross-indication meta-analyses of treatment-by-genotype interaction GWASs for primary (disease activity) outcomes
p values are from linear regression. The blue line shows genome-wide significance ($p = 5 \times 10^{-8}$) and the red line shows analysis-wide significance controlling for 19 analyses ($p = 2.63 \times 10^{-9}$, i.e., correcting for all individual GWASs and GWAS meta-analyses).

There are some limitations to our study that mean certain scenarios cannot be ruled out. Our use of genotyping arrays rather than genome sequencing meant we were unable to detect rare variants, so it is possible that lower-frequency variants of large effect may predict drug response. In addition, the wide confidence intervals on our SNP heritability estimates mean that we cannot rule out a moderate polygenic component consisting of a large number of variants of very small effect. Given our analysis of both single-disease and shared PRSs, if a polygenic component of drug response does exist, we posit that it would be uncorrelated with the genetics of disease susceptibility. This has been noted in other contexts, including the observation that genetic predictors of elevated low-density lipoprotein (LDL) capture the prognostic but not predictive response to LDL-lowering treatment and has motivated the development of direct pharmacogenomic PRS (PGx PRS) techniques.[37] Our study was also limited

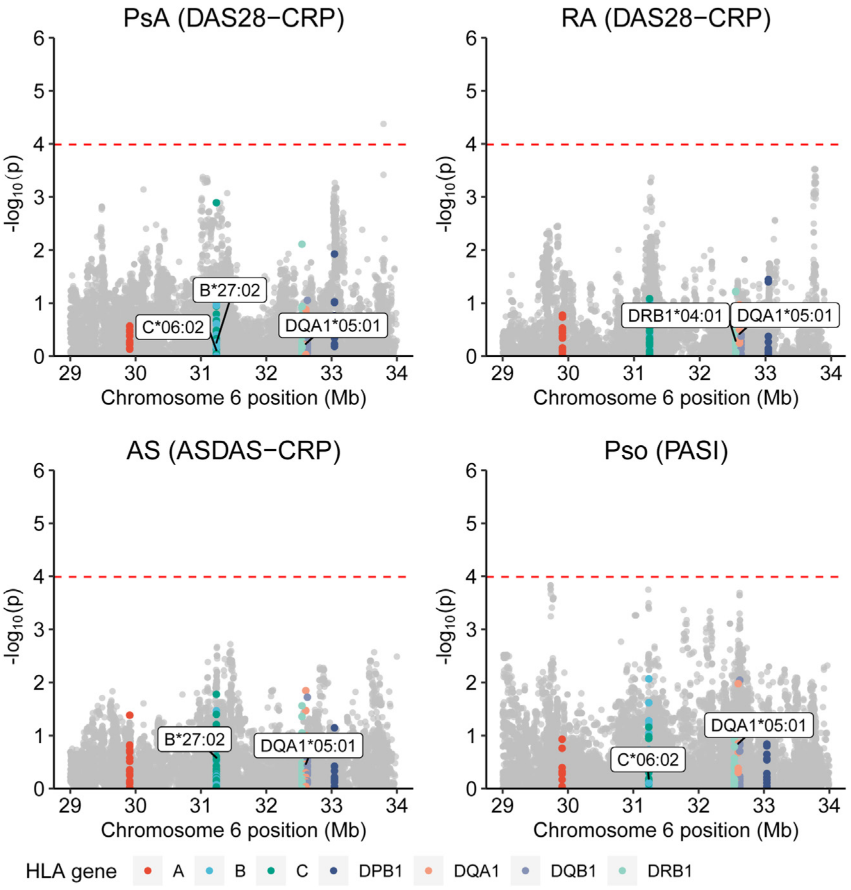

Figure 3. Regional plots of treatment-by-genotype interaction analysis for SNPs and HLA alleles in the HLA region for the primary outcome of each of the four indications tested

p values are from linear regression. Gray dots are SNPs and colored dotted are HLA alleles. The red dashed line shows analysis-wide significance for HLA alleles ($p = 1.02 \times 10^{-4}$), correcting for 489 tests of HLA alleles in four indications. Major susceptibility alleles for each indication are highlighted. Mb, Megabases.

by the outcomes collected, as past research has found that both disease activity and biomarker data are only incompletely correlated with imaging-based outcomes.[38] It remains possible that genetics may influence components of disease response not well captured by traditional clinical trial outcomes. Our sample size was also limited for some indications, so while we had high power for some analyses (particularly PsA and cross-indication outcomes), highly disease-specific gene-by-treatment interactions could have been missed for some indications (AS and RA, where N < 1000). Finally, some of our indications showed test-statistic inflation (as discussed in supplemental methods), which we attribute to model misspecification due to heteroscedasticity and to low sample size and minor allele frequency in certain smaller PC ancestry groups. Filtering and changes in model choice largely removed this inflation; however, it remains possible that some polygenic signal may also contribute to this inflation signal.

Secukinumab directly targets a pathway that has been repeatedly implicated in the genetics of inflammatory diseases,[39–43] including risk variants in genes both upstream of IL-17 production (*IL23R*, *TYK2*, *JAK2*, *STAT3*) and downstream of IL-17 response (*TRAF3IP2*/ACT1, *TNFAIP3*/A20). While genetic variation in this pathway impacts disease susceptibility, this variation does not seem to significantly influence response to treatment of that same pathogenic pathway. This may also have implications for hypotheses about endotypes in inflammatory disease, as genetically distinct participant subgroups with a strong differential response to treatment would manifest as genetic predictors of disease response in our study.

We believe that these results have important implications for future genetic studies of biological therapy in inflammatory disease and for the field of pharmacogenetics. Secukinumab has a large treatment effect while still maintaining significant unexplained variation in response, and this study was well powered and covered a variety of indications, outcomes, and ancestry groups. This suggests that, even in favorable conditions, finding predictive genetic biomarkers of response can prove difficult. While we do not yet know how widely this generalizes to other treatments and pathways, we should consider the possibility that genetics may not play a major role in response to other biological therapies as well, or that much larger sample sizes than can be acquired in even large clinical trials will be required to map small effect variants. Large cohorts of real-world participants undergoing routine treatment, such as the IBD BioResource[44] or HIPPOCRATES consortium,[45] may provide sample sizes required for mapping such variants, though these raise caveats around the lower precision of real-world outcomes and lack of randomized placebo control in real-world studies. It is likely that leveraging the advantages of both clinical trial and real-world data will be required to finally map the genetics of response to biological therapy while distinguishing predictive and prognostic biomarkers. We hope that this study will be a starting point for larger meta-analyses of the genetics of treatment response for inflammatory and autoimmune diseases.

Data and code availability

Anonymized clinical trial data are available upon request through Novartis' voluntary data-sharing process on ClinicalStudyData

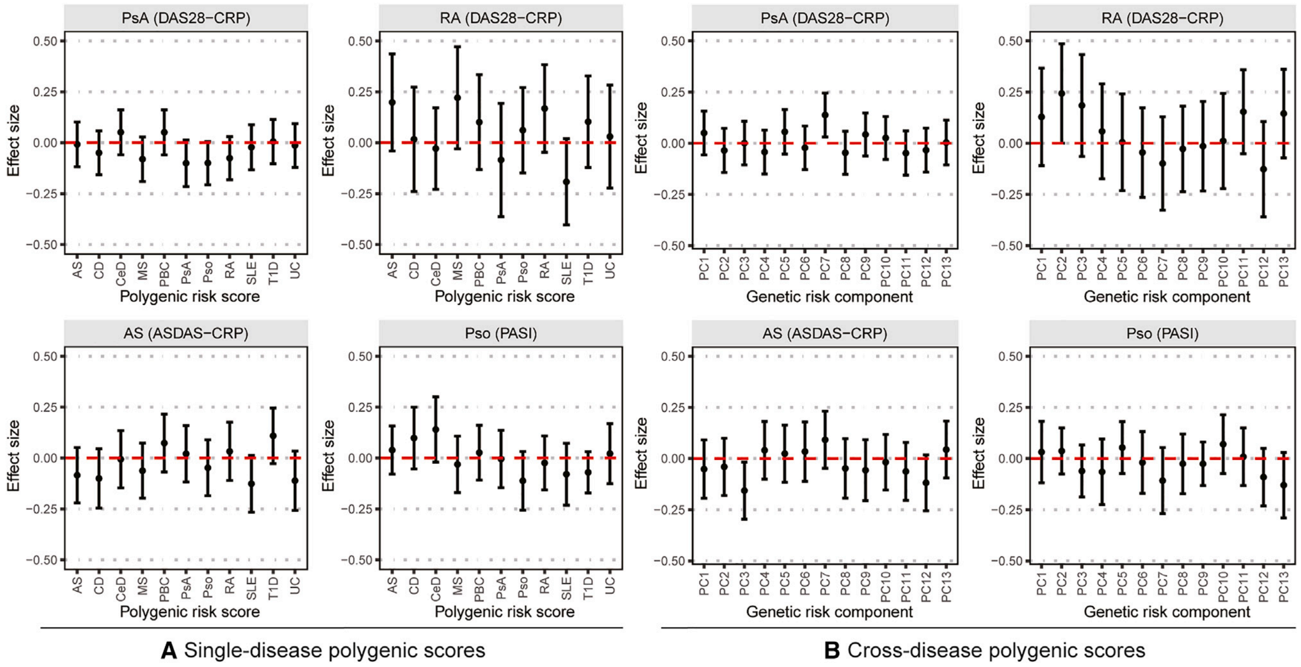

Figure 4. Interaction effect size between treatment and single disease or cross-disease polygenic risk scores on primary outcomes for the four indications
Effect sizes are given per standard deviation of the polygenic score. Error bars are 95% confidence intervals. Red dashed line corresponds to effect size = 0.

Request.com. Inquiries to access subject-level genetic data can be made through ClinicalStudyDataRequest.com and require a Sponsor Data-Sharing Agreement with Novartis Pharma AG. Full genome-wide summary statistics are available on the EBI GWAS Catalog (https://www.ebi.ac.uk/gwas/, accession numbers GCST90274734 to GCST90274752).

Supplemental information

Supplemental information can be found online at https://doi.org/10.1016/j.ajhg.2023.08.010.

Acknowledgments

The authors thank Anna Zalevski and Karine Lheritier for their ongoing support of this collaboration and Alain Marti for providing valuable feedback on the manuscript. L.J.-D. and B.H. were funded by Wellcome Trust grant 208750/Z/17/Z. The authors thank all participants in the clinical trials used in this study, without whom this research would not be possible.

Author contributions

C.Z., K.S., A.R., R.M., T.N., M.B., J.Z., E.F., M.V., and L.J.-D. designed the study. C.K., B.H., C.H., M.W., N.H., C.T., L.S., D.W., S.G., and A.-M.M. collected and processed the data. C.Z., K.S., B.H., J.Z., and L.J.-D. carried out the data analysis, and K.S., J.Z., E.F., M.V., and L.J.-D. guided the data analysis and interpretation. C.Z., B.H., and L.J.-D. produced the tables and figures. C.Z., K.S., B.H., M.B., J.Z., E.F., M.V., and L.J.-D. wrote the manuscript. All authors read and approved the manuscript.

Declaration of interests

C.Z., C.H., N.H., C.T., M.W., A.R., R.M., M.T.B., J.Z., E.F., and M.V. are employees and stock owners of Novartis AG or its subsidiaries. Other authors, including K.S., C.H.K., L.S., D.W., and S.G. were funded by the BDI-Novartis Collaboration for AI in Medicine, which is funded by a grant from Novartis AG. Novartis AG manufactures and markets secukinumab and has an ongoing commercial interest in its success.

Received: March 23, 2023
Accepted: August 15, 2023
Published: September 1, 2023

References

1. Roda, G., Jharap, B., Neeraj, N., and Colombel, J.-F. (2016). Loss of Response to Anti-TNFs: Definition, Epidemiology, and Management. Clin. Transl. Gastroenterol. *7*, e135.
2. Curry, P.D.K., Morris, A.P., Barton, A., and Bluett, J. (2023). Do genetics contribute to TNF inhibitor response prediction in Psoriatic Arthritis? Pharmacogenomics J. *23*, 1–7.
3. Hartl, D., de Luca, V., Kostikova, A., Laramie, J., Kennedy, S., Ferrero, E., Siegel, R., Fink, M., Ahmed, S., Millholland, J., et al. (2021). Translational precision medicine: an industry perspective. J. Transl. Med. *19*, 245.
4. Lin, C.M.A., Cooles, F.A.H., and Isaacs, J.D. (2022). Precision medicine: the precision gap in rheumatic disease. Nat. Rev. Rheumatol. *18*, 725–733.
5. Fahed, A.C., Philippakis, A.A., and Khera, A.V. (2022). The potential of polygenic scores to improve cost and efficiency of clinical trials. Nat. Commun. *13*, 2922.

6. Lewis, C.M., and Vassos, E. (2020). Polygenic risk scores: from research tools to clinical instruments. Genome Med. *12*, 44.
7. Meerman, J.J., Ter Hark, S.E., Janzing, J.G.E., and Coenen, M.J.H. (2022). The Potential of Polygenic Risk Scores to Predict Antidepressant Treatment Response in Major Depression: A Systematic Review. J. Affect. Disord. *304*, 1–11.
8. Liu, T., Li, S., Ying, S., Tang, S., Ding, Y., Li, Y., Qiao, J., and Fang, H. (2020). The IL-23/IL-17 Pathway in Inflammatory Skin Diseases: From Bench to Bedside. Front. Immunol. *11*, 594735.
9. Bianchi, E., and Rogge, L. (2019). The IL-23/IL-17 pathway in human chronic inflammatory diseases - new insight from genetics and targeted therapies. Microbes Infect. *21*, 246–253.
10. Parkes, M., Cortes, A., van Heel, D.A., and Brown, M.A. (2013). Genetic insights into common pathways and complex relationships among immune-mediated diseases. Nat. Rev. Genet. *14*, 661–673.
11. Burren, O.S., Reales, G., Wong, L., Bowes, J., Lee, J.C., Barton, A., Lyons, P.A., Smith, K.G.C., Thomson, W., Kirk, P.D.W., and Wallace, C. (2020). Genetic feature engineering enables characterisation of shared risk factors in immune-mediated diseases. Genome Med. *12*, 106.
12. Umi·evi· Mirkov, M., Cui, J., Vermeulen, S.H., Stahl, E.A., Toonen, E.J.M., Makkinje, R.R., Lee, A.T., Huizinga, T.W.J., Allaart, R., Barton, A., et al. (2013). Genome-wide association analysis of anti-TNF drug response in patients with rheumatoid arthritis. Ann. Rheum. Dis. *72*, 1375–1381.
13. Massey, J., Plant, D., Hyrich, K., Morgan, A.W., Wilson, A.G., Spiliopoulou, A., Colombo, M., McKeigue, P., Isaacs, J., Cordell, H., et al. (2018). Genome-wide association study of response to tumour necrosis factor inhibitor therapy in rheumatoid arthritis. Pharmacogenomics J. *18*, 657–664.
14. Sazonovs, A., Kennedy, N.A., Moutsianas, L., Heap, G.A., Rice, D.L., Reppell, M., Bewshea, C.M., Chanchlani, N., Walker, G.J., Perry, M.H., et al. (2020). HLA-DQA1*05 Carriage Associated With Development of Anti-Drug Antibodies to Infliximab and Adalimumab in Patients With Crohn's Disease. Gastroenterology *158*, 189–199.
15. Hässler, S., Bachelet, D., Duhaze, J., Szely, N., Gleizes, A., Hacein-Bey Abina, S., Aktas, O., Auer, M., Avouac, J., Birchler, M., et al. (2020). Clinicogenomic factors of biotherapy immunogenicity in autoimmune disease: A prospective multicohort study of the ABIRISK consortium. PLoS Med. *17*, e1003348.
16. Liu, M., Degner, J., Davis, J.W., Idler, K.B., Nader, A., Mostafa, N.M., and Waring, J.F. (2018). Identification of HLA-DRB1 association to adalimumab immunogenicity. PLoS One *13*, e0195325.
17. FDA-NIH Biomarker Working Group (2016). BEST (Biomarkers, Endpoints, and Other Tools) Resource. 2016 (US FDA). https://www.ncbi.nlm.nih.gov/books/NBK326791/.
18. Langley, R.G., Elewski, B.E., Lebwohl, M., Reich, K., Griffiths, C.E.M., Papp, K., Puig, L., Nakagawa, H., Spelman, L., Sigurgeirsson, B., et al. (2014). Secukinumab in plaque psoriasis–results of two phase 3 trials. N. Engl. J. Med. *371*, 326–338.
19. Mease, P., van der Heijde, D., Landewé, R., Mpofu, S., Rahman, P., Tahir, H., Singhal, A., Boettcher, E., Navarra, S., Meiser, K., et al. (2018). Secukinumab improves active psoriatic arthritis symptoms and inhibits radiographic progression: primary results from the randomised, double-blind, phase III FUTURE 5 study. Ann. Rheum. Dis. *77*, 890–897.
20. Pavelka, K., Kivitz, A., Dokoupilova, E., Blanco, R., Maradiaga, M., Tahir, H., Pricop, L., Andersson, M., Readie, A., and Porter, B. (2017). Efficacy, safety, and tolerability of secukinumab in patients with active ankylosing spondylitis: a randomized, double-blind phase 3 study, MEASURE 3. Arthritis Res. Ther. *19*, 285.
21. Blanco, F.J., Möricke, R., Dokoupilova, E., Codding, C., Neal, J., Andersson, M., Rohrer, S., and Richards, H. (2017). Secukinumab in active rheumatoid arthritis: A phase III randomized, double-blind, active comparator- and placebo-controlled study. Arthritis Rheumatol. *69*, 1144–1153.
22. Augustin, M., Jullien, D., Martin, A., and Peralta, C. (2020). Real-world evidence of secukinumab in psoriasis treatment - a meta-analysis of 43 studies. J. Eur. Acad. Dermatol. Venereol. *34*, 1174–1185.
23. Burmester, G.R., Durez, P., Shestakova, G., Genovese, M.C., Schulze-Koops, H., Li, Y., Wang, Y.A., Lewitzky, S., Koroleva, I., Berneis, A.A., et al. (2016). Association of HLA-DRB1 alleles with clinical responses to the anti-interleukin-17A monoclonal antibody secukinumab in active rheumatoid arthritis. Rheumatology *55*, 49–55.
24. Ramonda, R., Lorenzin, M., Sole Chimenti, M., D'Angelo, S., Marchesoni, A., Salvarani, C., Lubrano, E., Costa, L., Dal Bosco, Y., Fracassi, E., et al. (2022). Effectiveness and safety of secukinumab in axial spondyloarthritis: a 24-month prospective, multicenter real-life study. Ther. Adv. Musculoskelet. Dis. *14*. 1759720X221090310.
25. Costanzo, A., Bianchi, L., Flori, M.L., Malara, G., Stingeni, L., Bartezaghi, M., Carraro, L., Castellino, G.; and SUPREME Study Group (2018). Secukinumab shows high efficacy irrespective of HLA-Cw6 status in patients with moderate-to-severe plaque-type psoriasis: SUPREME study. Br. J. Dermatol. *179*, 1072–1080.
26. Morelli, M., Galluzzo, M., Madonna, S., Scarponi, C., Scaglione, G.L., Galluccio, T., Andreani, M., Pallotta, S., Girolomoni, G., Bianchi, L., et al. (2021). HLA-Cw6 and other HLA-C alleles, as well as MICB-DT, DDX58, and TYK2 genetic variants associate with optimal response to anti-IL-17A treatment in patients with psoriasis. Expert Opin. Biol. Ther. *21*, 259–270.
27. van der Heijde, D.M., van 't Hof, M.A., van Riel, P.L., Theunisse, L.A., Lubberts, E.W., van Leeuwen, M.A., van Rijswijk, M.H., and van de Putte, L.B. (1990). Judging disease activity in clinical practice in rheumatoid arthritis: first step in the development of a disease activity score. Ann. Rheum. Dis. *49*, 916–920.
28. Creemers, M.C.W., Van T Hof, M.A., Franssen, M.J.A.M., Van de Putte, L.B.A., Gribnau, F.W.J., and Van Riel, P.L.C.M. (1996). DISEASE ACTIVITY IN ANKYLOSING SPONDYLTTIS: SELECTION OF A CORE SET OF VARIABLES AND A FIRST STEP IN THE DEVELOPMENT OF A DISEASE ACTIVITY SCORE. Rheumatology *35*, 867–873.
29. Fredriksson, T., and Pettersson, U. (1978). Severe psoriasis–oral therapy with a new retinoid. Dermatology *157*, 238–244.
30. 1000 Genomes Project Consortium, Auton, A., Brooks, L.D., Durbin, R.M., Garrison, E.P., Kang, H.M., Korbel, J.O., Marchini, J.L., McCarthy, S., McVean, G.A., and Abecasis, G.R. (2015). A global reference for human genetic variation. Nature *526*, 68–74.
31. Rao, T.J., and Province, M.A. (2016). A framework for interpreting type I error rates from a product-term model of

interaction applied to quantitative traits. Genet. Epidemiol. *40*, 144–153.

32. Voorman, A., Lumley, T., McKnight, B., and Rice, K. (2011). Behavior of QQ-plots and genomic control in studies of gene-environment interaction. PLoS One *6*, e19416.
33. Cribari-Neto, F. (2004). Asymptotic inference under heteroskedasticity of unknown form. Comput. Stat. Data Anal. *45*, 215–233.
34. Bulik-Sullivan, B.K., Loh, P.-R., Finucane, H.K., Ripke, S., Yang, J., Schizophrenia Working Group of the Psychiatric Genomics Consortium, Patterson, N., Daly, M.J., Price, A.L., and Neale, B.M. (2015). LD Score regression distinguishes confounding from polygenicity in genome-wide association studies. Nat. Genet. *47*, 291–295.
35. Shin, J., and Lee, S.H. (2021). GxEsum: a novel approach to estimate the phenotypic variance explained by genome-wide GxE interaction based on GWAS summary statistics for biobank-scale data. Genome Biol. *22*, 183.
36. Kraft, P., Yen, Y.-C., Stram, D.O., Morrison, J., and Gauderman, W.J. (2007). Exploiting gene-environment interaction to detect genetic associations. Hum. Hered. *63*, 111–119.
37. Zhai, S., Zhang, H., Mehrotra, D.V., and Shen, J. (2022). Pharmacogenomics polygenic risk score for drug response prediction using PRS-PGx methods. Nat. Commun. *13*, 5278.
38. Østergaard, M., Bird, P., Pachai, C., Du, S., Wu, C., Landis, J., Fuerst, T., Ahmad, H.A., Connolly, S.E., and Conaghan, P.G. (2022). Implementation of the OMERACT Psoriatic Arthritis Magnetic Resonance Imaging Scoring System in a randomized phase IIb study of abatacept in psoriatic arthritis. Rheumatology *61*, 4305–4313.
39. Stuart, P.E., Nair, R.P., Tsoi, L.C., Tejasvi, T., Das, S., Kang, H.M., Ellinghaus, E., Chandran, V., Callis-Duffin, K., Ike, R., et al. (2015). Genome-wide Association Analysis of Psoriatic Arthritis and Cutaneous Psoriasis Reveals Differences in Their Genetic Architecture. Am. J. Hum. Genet. *97*, 816–836.
40. Tsoi, L.C., Stuart, P.E., Tian, C., Gudjonsson, J.E., Das, S., Zawistowski, M., Ellinghaus, E., Barker, J.N., Chandran, V., Dand, N., et al. (2017). Large scale meta-analysis characterizes genetic architecture for common psoriasis associated variants. Nat. Commun. *8*, 15382.
41. Tsoi, L.C., Spain, S.L., Knight, J., Ellinghaus, E., Stuart, P.E., Capon, F., Ding, J., Li, Y., Tejasvi, T., Gudjonsson, J.E., et al. (2012). Identification of 15 new psoriasis susceptibility loci highlights the role of innate immunity. Nat. Genet. *44*, 1341–1348.
42. Ha, E., Bae, S.-C., and Kim, K. (2021). Large-scale meta-analysis across East Asian and European populations updated genetic architecture and variant-driven biology of rheumatoid arthritis, identifying 11 novel susceptibility loci. Ann. Rheum. Dis. *80*, 558–565.
43. International Genetics of Ankylosing Spondylitis Consortium IGAS, Cortes, A., Hadler, J., Pointon, J.P., Robinson, P.C., Karaderi, T., Leo, P., Cremin, K., Pryce, K., Harris, J., et al. (2013). Identification of multiple risk variants for ankylosing spondylitis through high-density genotyping of immune-related loci. Nat. Genet. *45*, 730–738.
44. Parkes, M.; and IBD BioResource Investigators (2019). IBD BioResource: an open-access platform of 25 000 patients to accelerate research in Crohn's and Colitis. Gut *68*, 1537–1540.
45. FitzGerald, O., and Pennington, S.R. (2022). HIPPOCRATES: improving diagnosis and outcomes in psoriatic arthritis. Nat. Rev. Rheumatol. *18*, 123–124.

The American Journal of Human Genetics, Volume *110*

Supplemental information

Response to anti-IL17 therapy in inflammatory disease is not strongly impacted by genetic background

Cong Zhang, Konstantin Shestopaloff, Benjamin Hollis, Chun Hei Kwok, Claudia Hon, Nicole Hartmann, Chengeng Tian, Magdalena Wozniak, Luis Santos, Dominique West, Stephen Gardiner, Ann-Marie Mallon, Aimee Readie, Ruvie Martin, Thomas Nichols, Michael T. Beste, Jonas Zierer, Enrico Ferrero, Marc Vandemeulebroecke, and Luke Jostins-Dean

Supplemental Figures

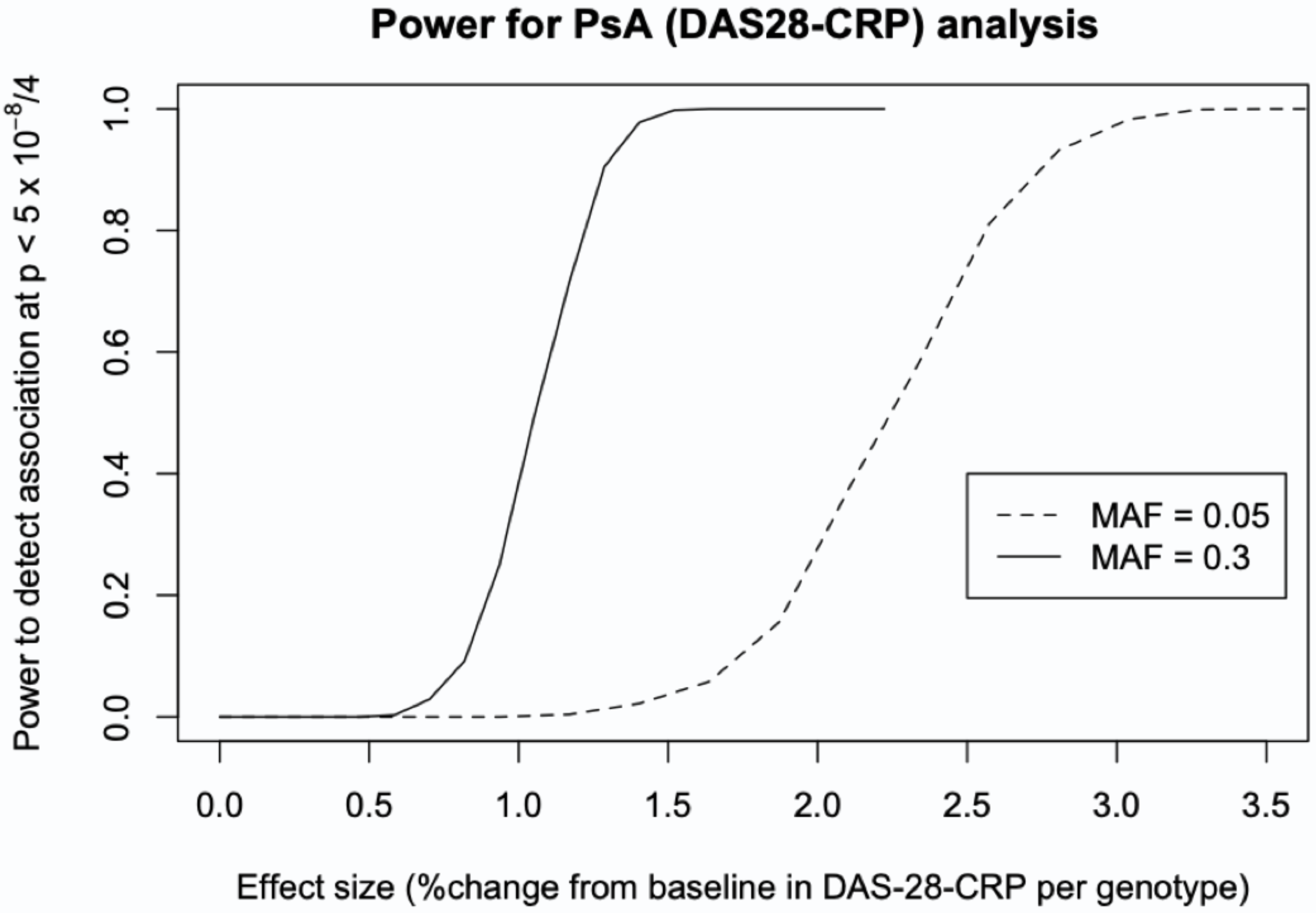

Figure S1: Power curves for a genome-wide genotype-by-treatment interaction for the largest indication/endpoint combination (PsA DAS28-CRP), for a lower frequency (MAF = 5%) and higher frequency (MAF = 30%) variant. MAF = Minor Allele Frequency.

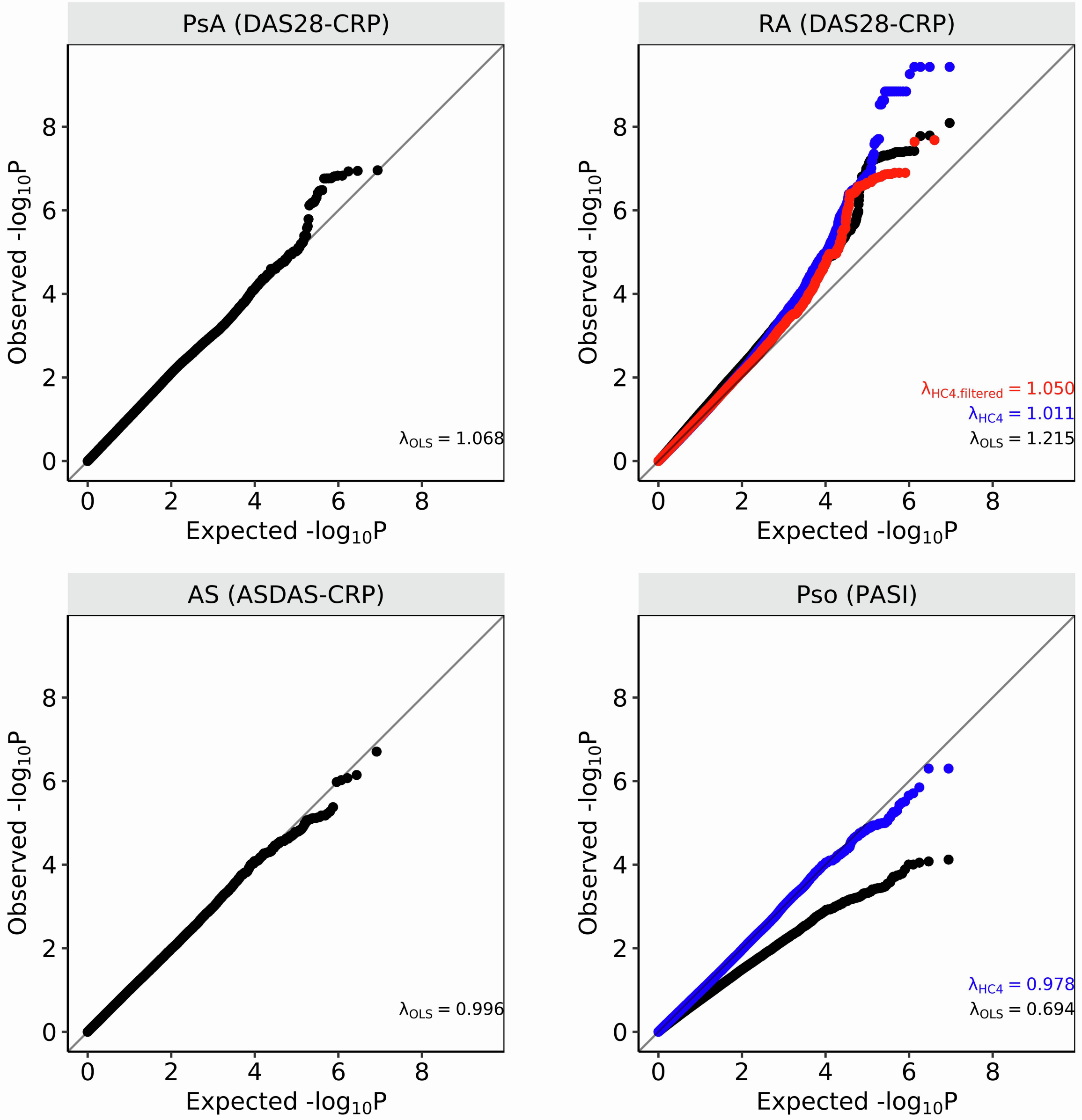

Figure S2: QQ plots for the treatment-by—genotype interaction GWAS for each of the primary outcomes for the four indications. Black dots shows the OLS regression result. The coloured dots show analyses that were rejected due to inflated or deflated QQ plots (blue for robust regression result with HC4 estimator used for rejected OLS regression, red for filtered robust regression result with HC4 estimator used for rejected robust regression). The OLS regression for PsA and AS, the robust regression for Pso and the heterogeneity-filtered robust regression for RA were used in the paper.

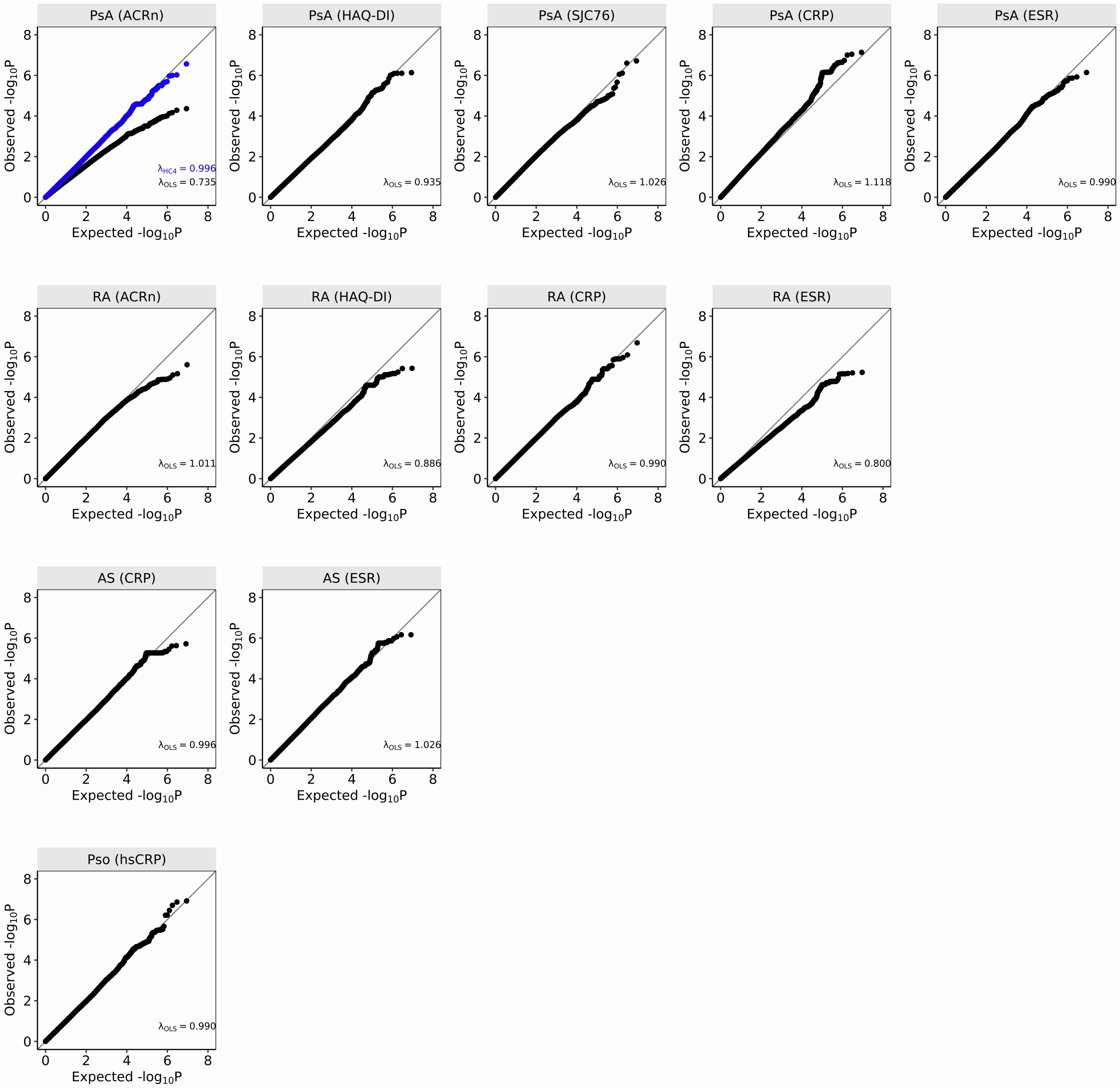

Figure S3: QQ plots for the treatment-by-genotype interaction GWAS for each of the secondary outcomes for the four indications. Black dots shows the OLS regression result. The coloured dots show analyses that were rejected due to inflated or deflated QQ plots (blue for robust regression result with HC4 estimator used for rejected OLS regression). The robust regression for PsA ACRn, OLS regression for all other outcomes were used in the paper.

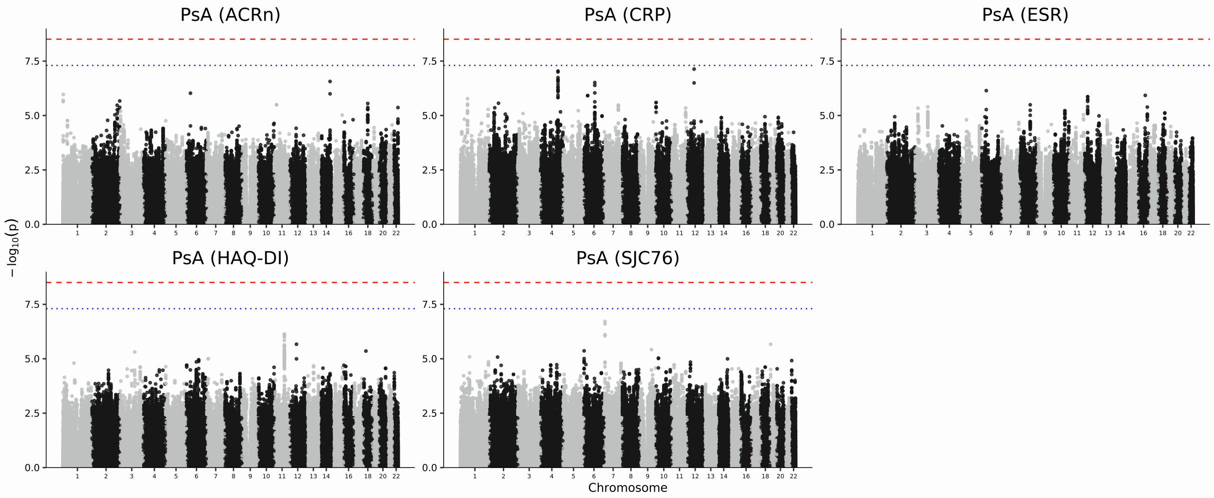

Figure S4: Manhattan plots of treatment-by-genotype interaction GWAS for the secondary outcomes for PsA. The blue line shows genome-wide significance (p = 5e-8) and the red line shows analysis-wide significance controlling for 16 analyses across all four indications (p = 3.12 x 10^{-9}).

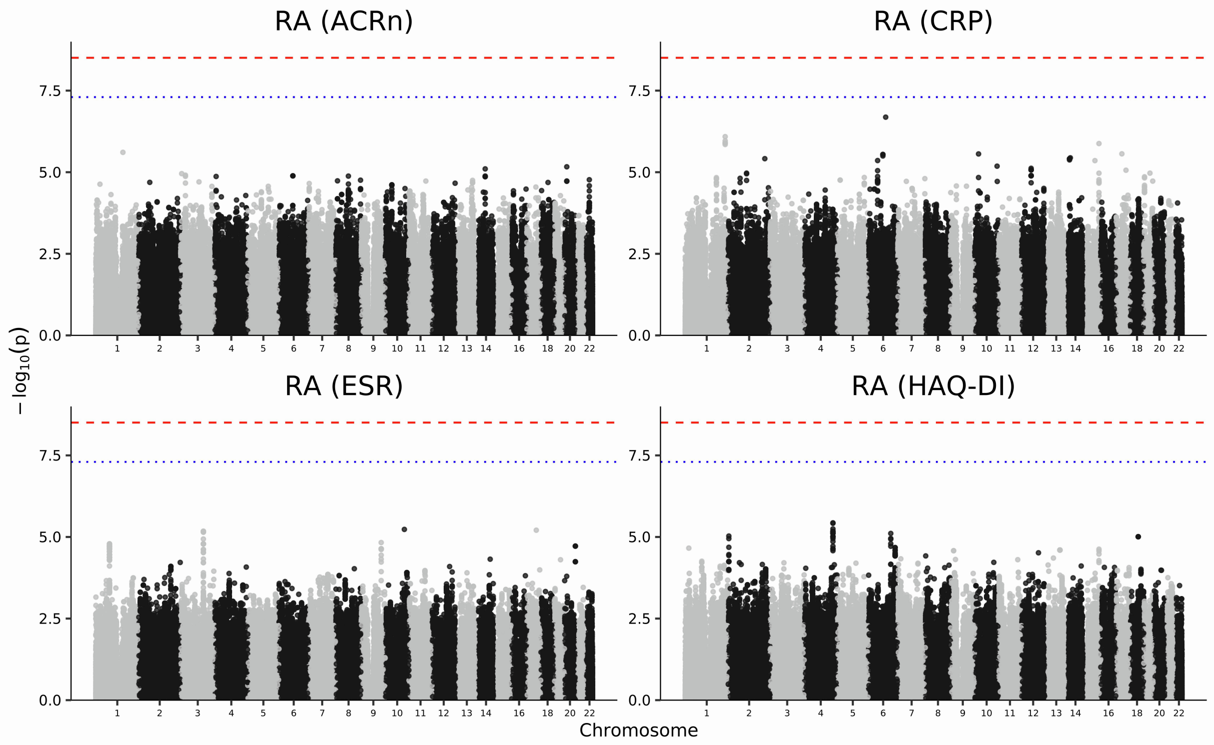

Figure S5: Manhattan plots of treatment-by-genotype interaction GWAS for the secondary outcomes for RA. The blue line shows genome-wide significance (p = 5 x 10^{-8}) and the red line shows analysis-wide significance controlling for 16 analyses across all four indications (p = 3.12 x 10^{-9}).

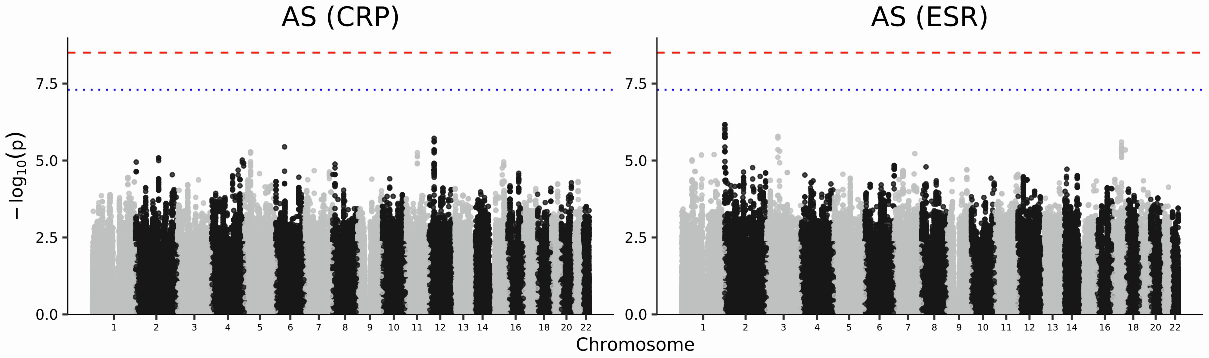

Figure S6: Manhattan plots of treatment-by-genotype interaction GWAS for the secondary outcomes for AS. The blue line shows genome-wide significance ($p = 5 \times 10^{-8}$) and the red line shows analysis-wide significance controlling for 16 analyses across all four indications ($p = 3.12 \times 10^{-9}$).

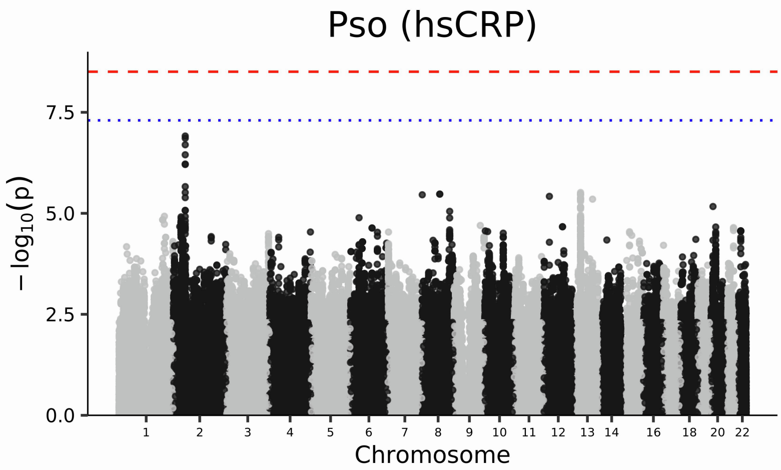

Figure S7: Manhattan plots of treatment-by-genotype interaction GWAS for the secondary outcomes for Pso. The blue line shows genome-wide significance ($p = 5 \times 10^{-8}$) and the red line shows analysis-wide significance controlling for 16 analyses across all four indications ($p = 3.12 \times 10^{-9}$).

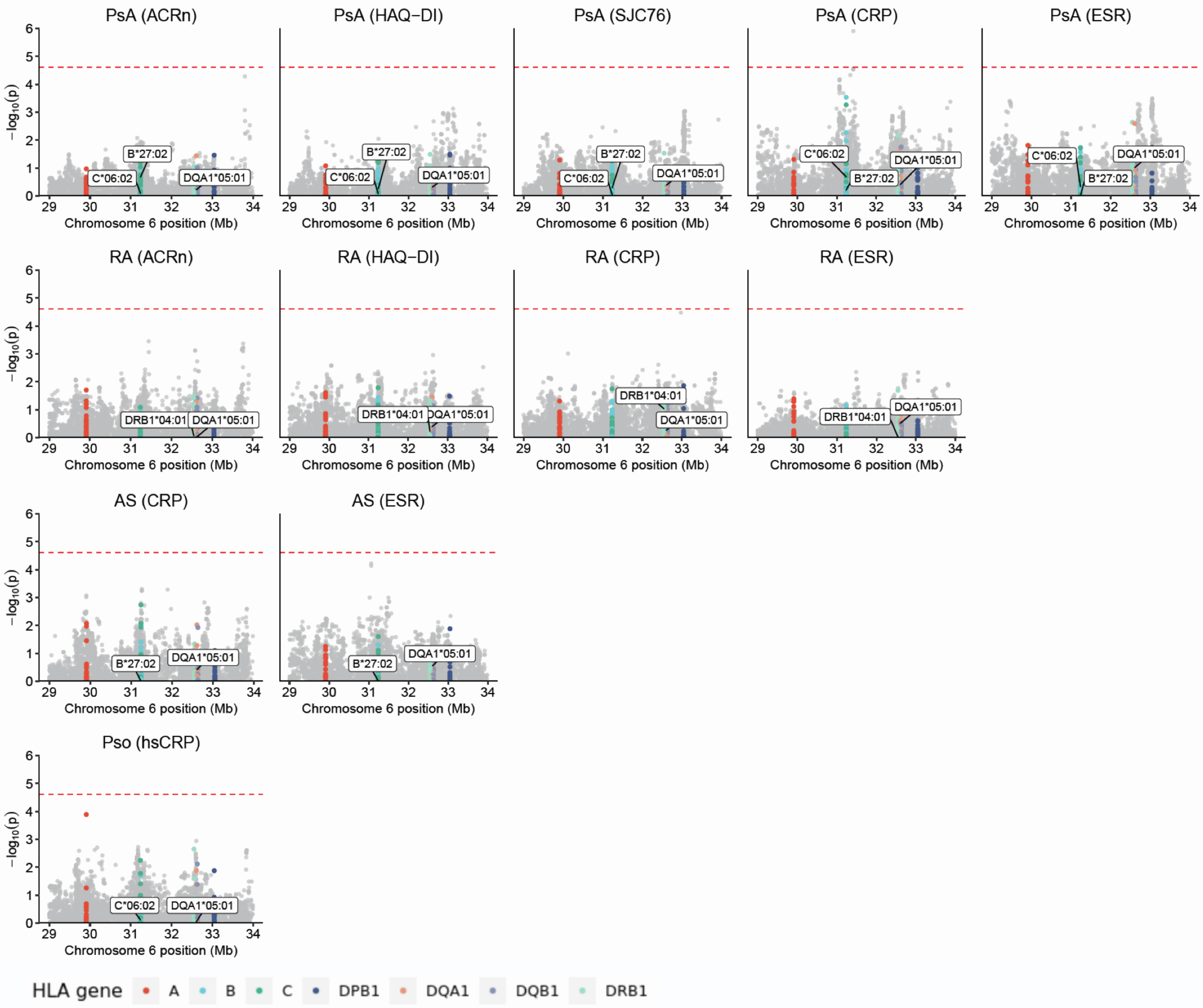

Figure S8: Regional plots of treatment-by-genotype interaction analysis for SNPs and HLA alleles in the HLA region for the secondary outcomes of each of the four indications tested (PsA, RA, AS and Pso). Grey dots are SNPs, and coloured dotted are HLA alleles. The red dashed line shows analysis-wide significance for HLA alleles ($p = 2.41 \times 10^{-5}$), correcting for a total of 2,074 tests of HLA alleles in 15 endpoints. Mb = Megabases.

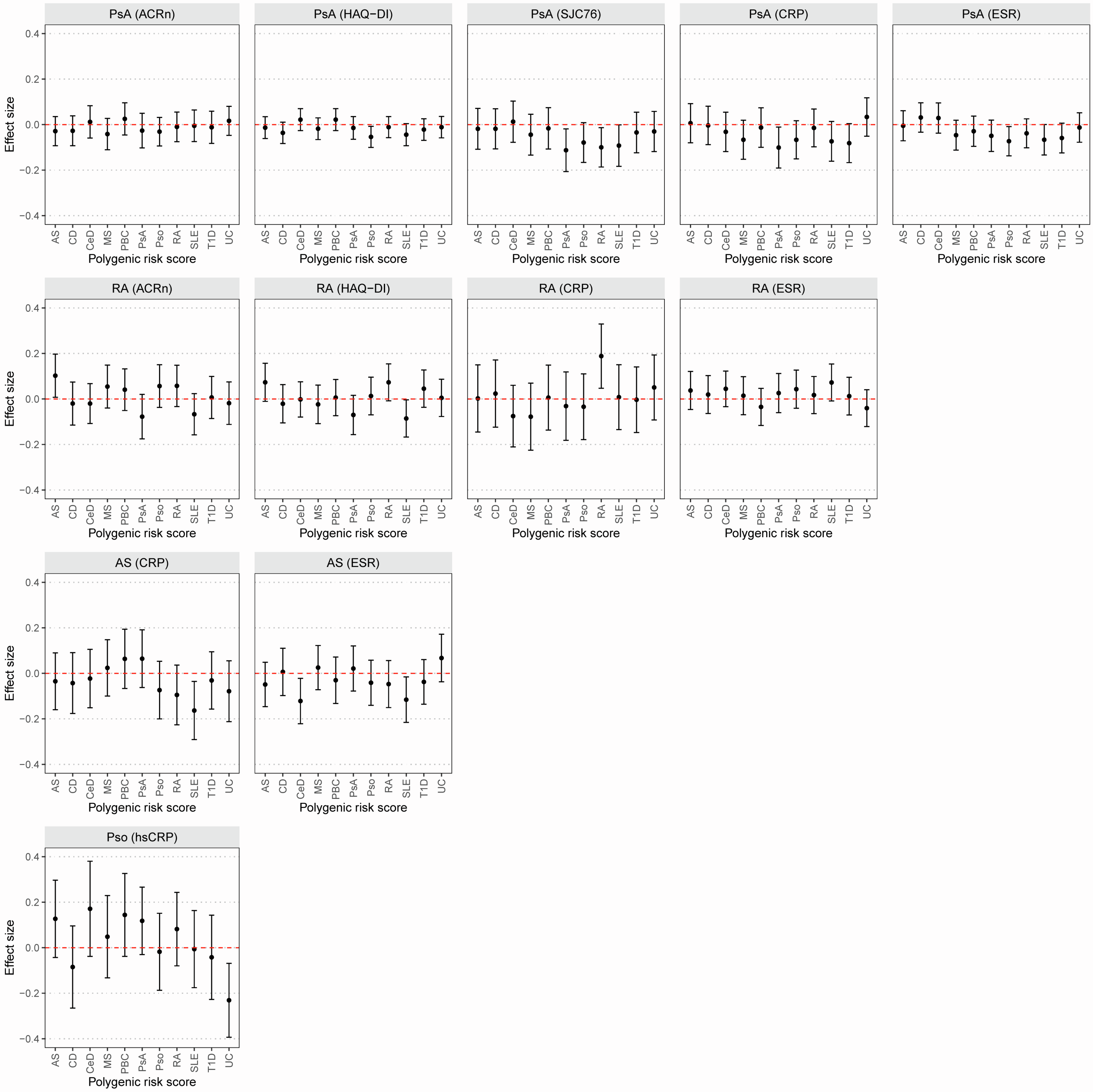

Figure S9: Interaction effect size between treatment and single disease polygenic risk scores on secondary outcomes for four indications. Effect sizes are given per standard deviation of the polygenic score. Error bars are 95% confidence intervals. Red dashed line corresponds to effect size = 0. Abbreviation of polygenic risk score traits: Ankylosing Spondylitis (AS), Celiac Disease (CeD), Crohn's Disease (CD), Multiple Sclerosis (MS), Primary Biliary Cirrhosis (PBC), Psoriatic Arthritis (PsA), Rheumatoid Arthritis (RA) Systemic Lupus Erythematosus (SLE), Type 1 Diabetes (T1D), Ulcerative Colitis (UC).

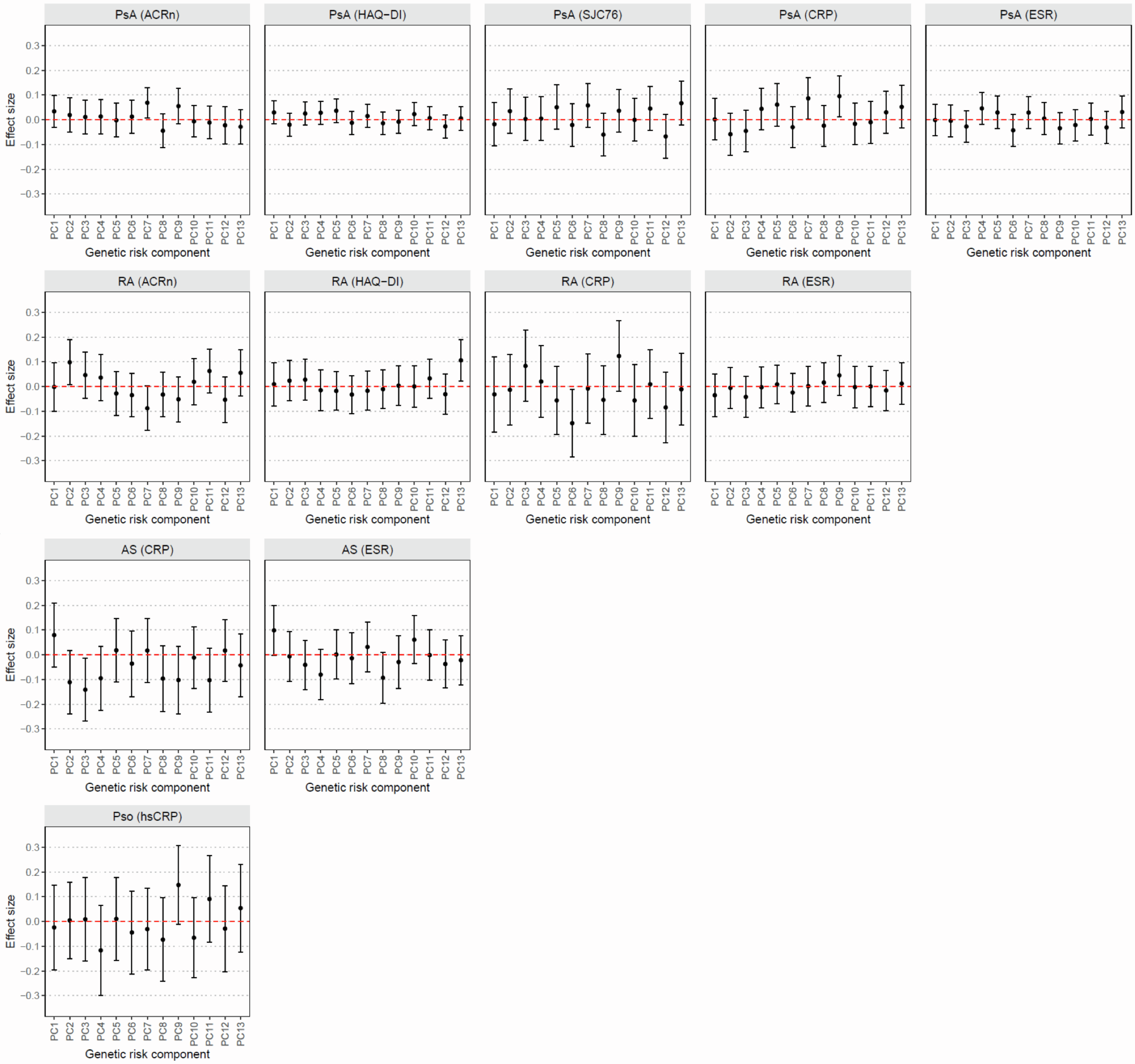

Figure S10: Interaction effect size between treatment and cross-trait polygenic risk scores on secondary outcomes for four indications. Effect sizes are given per standard deviation of the polygenic score. Error bars are 95% confidence intervals. Red dashed line corresponds to effect size = 0.

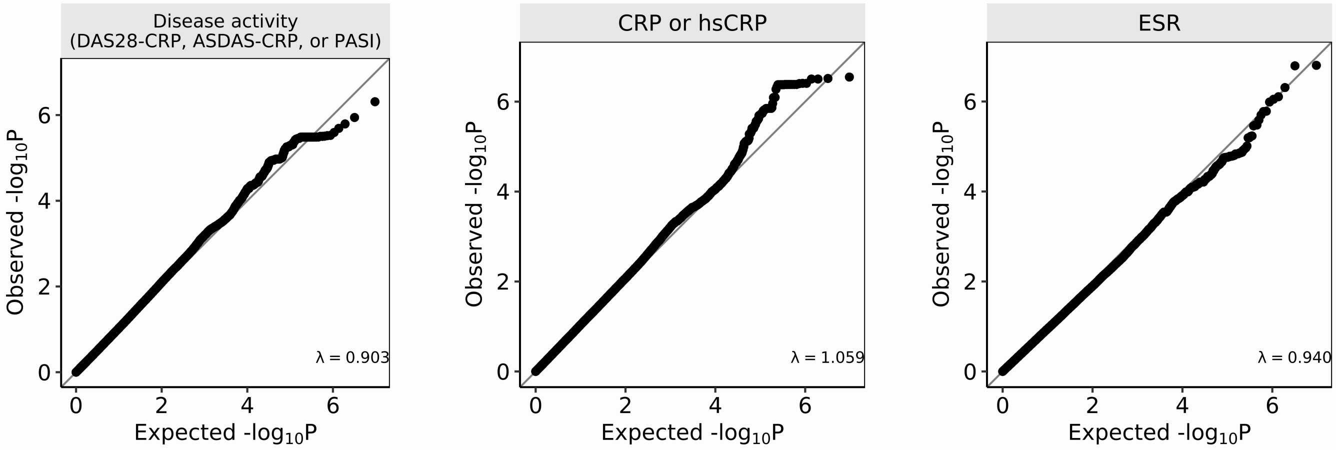

Figure S11: QQ plots for the cross-indication meta-analyses of treatment-by-genotype interaction GWAS for selected primary (disease activity) and secondary (CRP and ESR) outcomes.

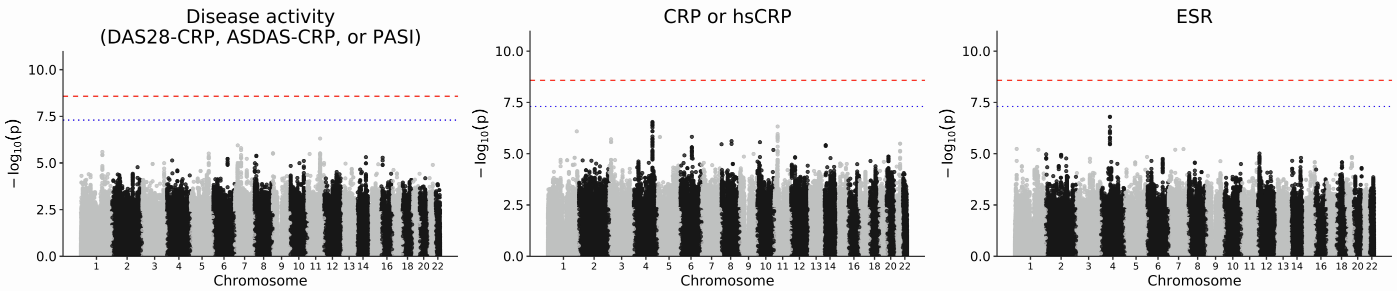

Figure S12: Manhattan plots of cross-indication meta-analyses of treatment-by-genotype interaction GWAS for selected primary (disease activity) and secondary (CRP and ESR) outcomes. The blue line shows genome-wide significance ($p = 5 \times 10^{-8}$) and the red line shows analysis-wide significance controlling for 19 analyses ($p = 2.63 \times 10^{-9}$, i.e. correcting for all individual GWAS and GWAS meta-analyses).

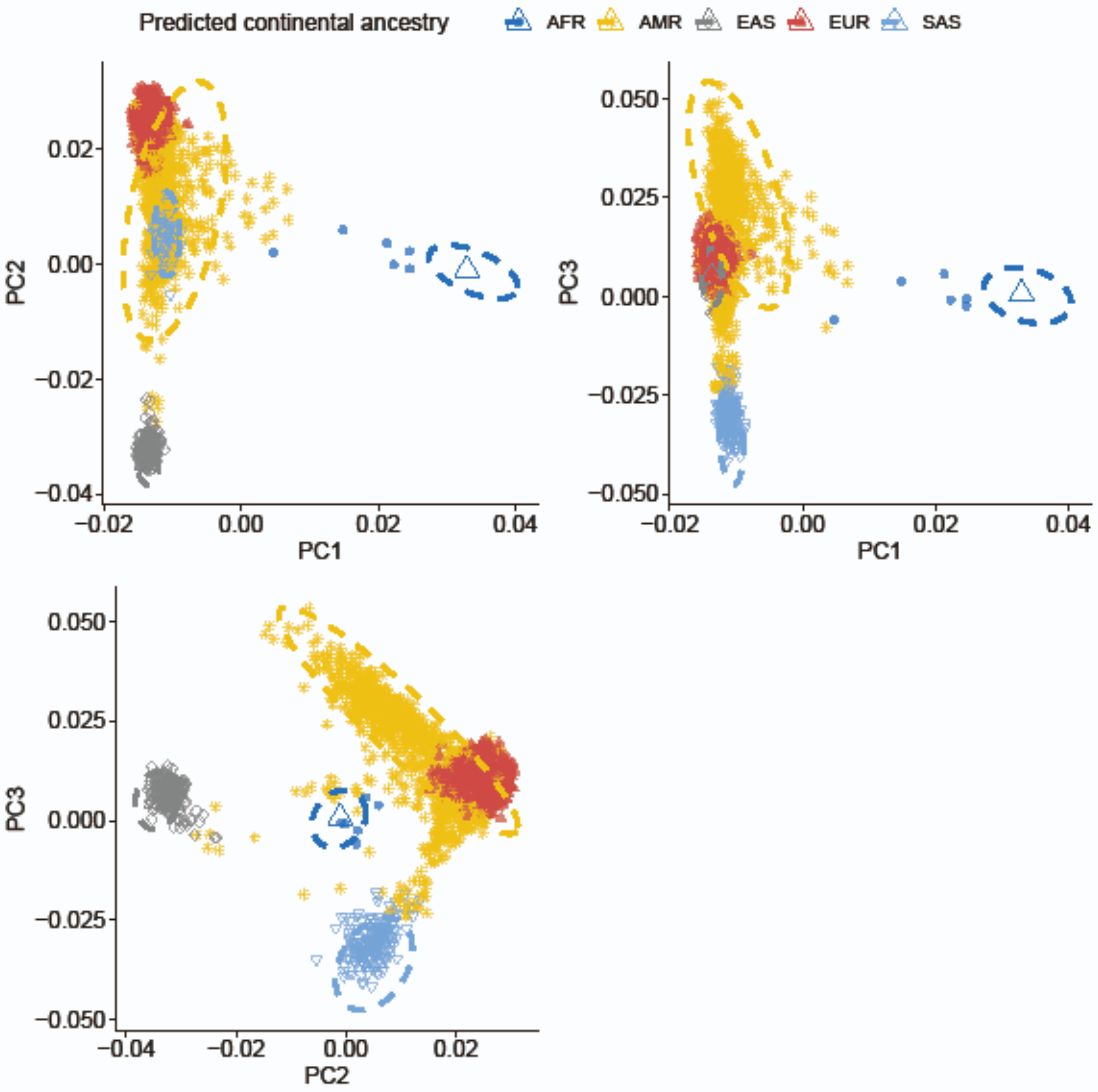

Figure S13: Ancestry assignment by principal component analysis. Each dot represents a patient sample with the color and shape indicating the predicted continental ancestry. The dashed ellipses showed the 95% confidence region of the 1k genome samples in the corresponding ethnic group .

Supplemental Methods

Ethics and consent
This was a post-hoc analysis of existing anonymized clinical trial data collected by Novartis Pharmaceuticals[1]. All trials included in the study are listed in Table S2, and this table includes NCT numbers to allow further information to be found via clinicaltrials.gov.

The site investigators collected the data, Novartis Pharmaceuticals and the University of Oxford conducted the data analyses, and all the authors had access to the data. The studies were approved by the institutional review board or ethics committee at each participating site, and the studies were conducted in accordance with the ethical principles of the Declaration of Helsinki. U.S. sites maintained compliance with Health Insurance Portability and Accountability Act regulations. Eligible patients provided written informed consent.

Power calculations
Power for the largest indication (psoriatic arthritis) was assessed by simulation. Datasets of patients with simulated changes in pre- and post-treatment DAS28-CRP levels were generated under a Gaussian linear model, with number of treatment and placebo cases taken from Table 1, a main treatment effect size of 0.76 units, a genotype-by-treatment interaction term of varying effect size, and a Gaussian error selected to maintain a standard deviation of 0.11 within treatment (all fixed parameters selected to match the previously reported results of study NCT02404350[2]). Power was assessed by the number of simulations that met the primary analysis p-value threshold ($p < 1.25 \times 10\text{-}8$) for a given interaction effect size.

Processing and quality control of genotype data
DNA genotyping and base calling were carried out separately for each study on the Illumina Global Screening Array and processed in GenomeStudio. Genotype data for each study underwent a standard quality control pipeline to detect duplicate samples (pi_hat > 0.9), poorly genotyped samples (missingness > 10%), poorly genotyped variants (missingness > 5%), and a pre-defined set of poorly performing probes provided by Illumina. Samples also underwent a sex check to detect mismatches between chromosomal sex and patient reported sex to detect probable sample swaps. Variants were then aligned to the forward strand based on allele complementary and allele frequencies, with ambiguous variants with a minor allele frequency > 20% excluded. Variants were then phased using SHAPEIT[3] (v2.r837) and imputed using IMPUTE2[4] (v2.3.2) with 1000 Genomes haplotypes used as the reference (Phase 3 b37[5]).

After imputation, we carried out another level of QC on each study. Variants with an info score of less than 0.7 or a minor allele frequency of less than 1% were removed. Finally, hard calls were generated by setting genotypes to their highest posterior value, and setting calls where the best guess posterior was less than 90% to missing. All samples with > 10 % missing data at SNPs with < 10% missing data within each study were removed from further analyses, as well as SNPs with a Hardy-Weinberg Equilibrium (HWE)-threshold of 1×10^{-6} or a minor allele frequency of less than 0.25 %. Related and duplicated samples (pi_hat > 0.125) were also filtered out. The individual studies for each indication were then merged into a

single file, and triallelic SNPs were removed, as were variants with a minor allele frequency <1% across an entire indication.

Studies were merged by indication and PCA ancestry (see the next section for a description of how PCA ancestry was assigned) group, and a final round of QC was performed within these groups. All variants with missing genotype rate > 2%, failing the HWE p-value-threshold of 1×10^{-6}, having a minor allele frequency of less than 1 %, or with significantly different allele frequencies across the batches based on a false-discovery rate (FDR) threshold of 1×10^{-5} for each test were removed. In addition, individuals with missing genotype rate >2 %, with inconsistent reported and genetically determined sex, or that had a significantly higher or lower inbreeding coefficient (F) (calculated using the '--het' option in plink) at FDR 0.05, showed a relatedness with pi_hat > 0.18, were removed.

PCA analysis and PCA ancestry assignment

Merged samples were classified into continental ancestry groups (which we refer to as "PCA ancestry") using principal component analysis (PCA) using a 1000 Genome reference set. More specifically, we carried out principal component analysis on the 1000 Genome phase 3 samples, using only those variants that were present in both 1000 Genome and the GSA chip. Each of our study samples was then projected onto the first two principal component axes, and for each sample we calculated a z-scores for each of the five 1000 Genomes continental groups (EUR/Europeans, AFR/Africans, SAS/South Asians, EAS/East Asians, AMR/Admixed Americas), based on the distance of the sample to the centre of the continental groups. Each sample was assigned to one of these five PCA ancestry groups according to the minimal z-score. The position of our samples in this PCA space, coloured by ancestry alignment, is shown in Supplementary Figure S13. PCA within each assigned ancestry group samples were also performed and the first three principal components were used to control for population stratification in the following analysis.

Imputation of common HLA alleles

Common HLA alleles were imputed to 4-digit resolution using the HIBAG package[6] (v3.12), using a prediction model built for the Global Screening Array. We imputed alleles separately within each PCA ancestry cluster, using the European model to predict EUR samples, the Asian model to predict SAS and EAS samples, the African model to predict AFR samples and the Hispanic model to predict AMR samples. Only calls with posterior probability >0.5 were included (otherwise the call was set to missing), and the maximum posterior allele assignment as used. HLA alleles were filtered to include only those with an allele frequency greater than 1% within each ancestry cluster, and a new variable for the dosage of each HLA allele were generated for downstream testing.

Association analysis and follow-up

A GWAS was run using plink (v1.9b) for each primary and secondary outcome within the four indication and across each PCA ancestry with at least 100 samples for that indication. The primary outcomes were disease activity scores (DAS28-CRP for RA and PsA, ASDAS-CRP for AS, PASI for Pso), and secondary outcomes included biomarkers (CRP, ESR for RA, PsA and AS as well as hsCRP for Pso), a drug response measure (ACRn, for RA and PsA), a measure of disability (HAQ-DI for RA and PsA) and a measure of swollen joint count of 76

joints (SJC76 for PsA, see table below for full description). For each endpoint, the GWAS was carried out separately within each PCA ancestry group with at least 100 samples, and then a combined result for that endpoint was generated by meta-analysing the results across PCA ancestry groups using a fixed effect inverse-variance weighted approach (with effect size estimates and standard errors as input). The sample-size cut-off of 100 as chosen to prevent false positives due to underestimated standard errors, which are common with smaller sample sizes [7]. Three outcomes (CRP, ESR and disease activity scores) were also meta-analyzed across indications using a sample-size-weighted approach (with p-value and direction of effect as input, weighted by sample size) to account for the possibility of differences in effect size across indications. All meta-analyses were calculated using the program METAL[8]. Prior to analysis, PASI scores were transformed to normal quantiles, biomarkers (CRP, hsCRP and ESR) were both log(x+1) transformed, and ACRn was transformed using log[(100 - x) + 1]. A summary of the outcomes tested, with descriptions, ranges and transformations is below:

**Indication(s)**	**Outcome**	**Description**	**Primary/secondary**	**Range**	**Transformation**
PsA/RA	DAS28-CRP	Disease Activity Score-28 with CRP	Primary	0 – X*	None
PsA/RA	ACRn	% improvement in American College of Rheumatology multidimensional response score	Secondary	0 - 100	log[(100 - x) + 1]
PsA/RA/AS/Pso	CRP/hsCRP	Serum C-reactive protein measurement	Secondary	0 – X*	log(x + 1)
PsA/RA/AS	ESR	Blood erythrocyte sedimentation rate	Secondary	0 – X*	log(x + 1)
PsA/RA	HAQ-DI	Health Assessment Questionnaire Disability Index	Secondary	0 - 3	None
PsA	SJC76	Swollen Joint Count 76	Secondary	0 - 76	None
AS	ASDAS-CRP	Ankylosing Spondylitis Disease Activity Score with CRP	Primary	0 - X*	None
Pso	PASI	Psoriasis Area and Severity Index	Primary	0 - 72	Quantile normalized

*biomarker values are theoretically unbounded, values here are maximum in our data

Each GWAS assessed the effects of gene-treatment interaction on each outcome of interest at 16 weeks for PsA, RA and AS and at 12 weeks for Pso (i.e. the primary readout time-points in the original study protocols) using linear regression, adjusting for baseline measure (where available), demographics (age, sex and BMI categorized into four groups according

to the WHO classification[9]), the top 3 genetic PCs (selected based on the scree plots within each ethnicity), study population, use of methotrexate (MTX) and tumor necrosis factor inhibitor (TNFi) medications (where available), as well as SNP and treatment main effects. "Treatment" was defined as any patient randomized to reive secukinumab, regardless of dosing regimen. Subjects with outcome measures within 2 weeks of the target times were included in the analysis. All models were adjusted for baseline except ACRn, as it was used in the calculation of the outcome. The model fit for analysis was:

outcome_endpoint = outcome_baseline + age + sex + BMI + study + MTX + TNFi + PC1 + PC2 + PC3 + treatment + SNP + treatment × SNP

In addition, for all analyses we also recorded the summary statistics for the main effect (I.e. the coefficient for SNP in the above model), and the joint effect p-value (a hypothesis testing comparing whether either of the SNP or treatment × SNP effects are non-zero, as described in [10]).

Initially, all analyses were carried out in Plink using standard ordinary least squares (OLS) linear regression. However, it is known that model misspecification, and in particular heteroscedasticity (i.e. a violation of the homogeneity of variance assumption) can lead to false negatives and false positives in genetic interaction models [11, 12]. We tested all combinations of indication, outcome and ancestry group for heteroscedasticity under the null using the Breusch-Pagan test [13] and found significant deviation from homogeneity of variance in many of our models (Supplementary Table S11). We inspected -log10 QQ plots for all indication and outcomes (Supplementary Figures S2, S3 and S11), and saw evidence of significant inflation or deflation for three analyses (deflation in PsA ACRn and Pso PASI, and inflation for RA DAS28-CRP). We re-ran the full genome-wide association analyses for these three traits using the same model but with standard errors and p-value estimated using a heteroscedasticity robust HC4 sandwich estimator[14], implemented in the R package sandwich. This produced well-calibrated QQ plots for PsA ACRn and Pso PASI (black dots in Supplementary Figure S2 and S3). However, the QQ plot for RA DAS28CRP remained inflated even in the robust analysis. We carried out a permutation analysis to test if this inflation was caused by miscalibrated p-values under the null, by permuting the genotypes for the genome-wide-significant variants 100,000 times and refitting the robust regression test to generate an empirical null distribution. This analysis showed that the genome-wide significant associations in this analysis were false positive artefacts (having empirical permuted $p > 1e-5$). These false positives were driven by extremely significant results in a single ancestry group, so to remove them we filtered out results that had inconsistent associations across the ancestry groups by applying a filter for heterogeneity of effect ($p < 0.05$) using Cochran's Q[15]. This filtered dataset produced a well-calibrated QQ plot and removed the false positive artefacts, though we should note a limitation of this approach is that it may also remove truly population-specific associations as well. Our final set of summary statistics thus consisted of robust regression results for Pso PASI and PsA ACRn, robust regression results with heterogeneity of effect filtering for RA DAS28CRP, and OLS results for all other analyses. The final regression test selected for each outcome is shown in

Supplementary Table S3. We also tested all genome-wide significant associations from any analysis in both OLS and robust regression as a post-hoc sensitivity check.

We calculated the proportion of variance explained by treatment-gene interactions (the GxE heritability) using LDSC[16] with default parameters, as detailed by Shin *et al*[17].

Testing of polygenic risk scores for susceptibility

To produce a set of associations to immune or inflammatory diseases, for the purposes of calculating polygenic risk scores (PRS), 12 studies of immune mediated diseases with full summary statistics were identified from the EBI GWAS catalogue[18]. Information on studies included are in Supplementary Table S7. Summary statistics were imputed using the ssimp package[19] using ancestry-matched 1000 Genomes reference sets. We calculated polygenic risk scores using the genome-wide Bayesian shrinkage model PRS-CS[20], using the settings suggested in the tutorial (including setting the shrinkage parameter phi=1e-2, as suggested for smaller GWAS). The 12 scores were then calculated for each sample in our study using plink (v1.9b). We also calculated polygenic risk scores for the 1000 Genomes samples, and validated that the correct scores were associated with the correct indications (e.g. the PsA score was higher in the PsA indication than in the 1000 Genomes samples for the same PCA ancestry, data not shown).

Decomposed polygenic risk scores were calculated using betas for each of the 13 principal component basis of shared genetic risk for immune-mediated diseases identified by Burren *et al*[21]. We attempted to regenerate principal components for our 12 IMD studies using the same cupcake method, but this did not produce a sparse basis, likely due to the low sample sizes (Burren *et al* recommends N > 6000, which not all of our 12 studies met) and the fact that some of our studies were not genome-wide (as we included studies with targeted Immunochip data). We thus used the basis generated by Burren *et al*, as at least one of the 13 Burren *et al* components was associated with each of our four indications, suggesting that the genetic components identified covered the relevant region of genetic risk for our samples. We calculated polygenic scores for each sample in each of our four indications, using the approach described in Burren *et al*.

We assessed the effects of PRS-treatment interaction, as well as main and joint effects, on each outcome of interest for the four indications using linear model, adjusting for the same covariates in the GWAS model, including baseline measures, demographics (age, sex and WHO categories of BMI), Study ID, the top 3 genetic PCs, use of MTX, and TNFi medications (where available). The polygenic scores were normalized to zero-mean and unit-variance for comparison purpose. We used robust sandwich estimators for Pso PASI, RA DAS-28-CRP and PsA ACRn, to mirror the approach used for the GWAS analysis. The association model was run for each outcome within the four indication and across each ethnicity (European, Admixed American and East Asian) in populations with at least 100 samples. The results from the individual models were then meta-analyzed across ethnicity using a fixed-effect inverse-variance-weighted standard error approach. We carried out multiple testing correction using the Benjamini-Hochberg procedure to preserve a false discovery rate of 0.05.

Association testing of HLA alleles

The same approach to testing PRS-treatment interactions was also used to test HLA-treatment interactions.

Supplemental References:

1. Mallon, A.-M., Häring, D.A., Dahlke, F., Aarden, P., Afyouni, S., Delbarre, D., El Emam, K., Ganjgahi, H., Gardiner, S., Kwok, C.H., et al. (2021). Advancing data science in drug development through an innovative computational framework for data sharing and statistical analysis. BMC Med. Res. Methodol. *21*, 250.

2. Mease, P., van der Heijde, D., Landewé, R., Mpofu, S., Rahman, P., Tahir, H., Singhal, A., Boettcher, E., Navarra, S., Meiser, K., et al. (2018). Secukinumab improves active psoriatic arthritis symptoms and inhibits radiographic progression: primary results from the randomised, double-blind, phase III FUTURE 5 study. Ann. Rheum. Dis. *77*, 890–897.

3. Delaneau, O., Coulonges, C., and Zagury, J.-F. (2008). Shape-IT: new rapid and accurate algorithm for haplotype inference. BMC Bioinformatics *9*, 540.

4. Howie, B.N., Donnelly, P., and Marchini, J. (2009). A flexible and accurate genotype imputation method for the next generation of genome-wide association studies. PLoS Genet. *5*, e1000529.

5. 1000 Genomes Project Consortium, Auton, A., Brooks, L.D., Durbin, R.M., Garrison, E.P., Kang, H.M., Korbel, J.O., Marchini, J.L., McCarthy, S., McVean, G.A., et al. (2015). A global reference for human genetic variation. Nature *526*, 68–74.

6. Zheng, X., Shen, J., Cox, C., Wakefield, J.C., Ehm, M.G., Nelson, M.R., and Weir, B.S. (2014). HIBAG--HLA genotype imputation with attribute bagging. Pharmacogenomics J. *14*, 192–200.

7. Lin, L. (2018). Bias caused by sampling error in meta-analysis with small sample sizes. PLoS One *13*, e0204056.

8. Willer, C.J., Li, Y., and Abecasis, G.R. (2010). METAL: fast and efficient meta-analysis of genomewide association scans. Bioinformatics *26*, 2190–2191.

9. World Health Organization (2000). Obesity: Preventing and Managing the Global Epidemic : Report of a WHO Consultation (World Health Organization).

10. Kraft, P., Yen, Y.-C., Stram, D.O., Morrison, J., and Gauderman, W.J. (2007). Exploiting gene-environment interaction to detect genetic associations. Hum. Hered. *63*, 111–119.

11. Rao, T.J., and Province, M.A. (2016). A framework for interpreting type I error rates from a product-term model of interaction applied to quantitative traits. Genet. Epidemiol. *40*, 144–153.

12. Voorman, A., Lumley, T., McKnight, B., and Rice, K. (2011). Behavior of QQ-plots and genomic control in studies of gene-environment interaction. PLoS One *6*, e19416.

13. Breusch, T.S., and Pagan, A.R. (1979). A Simple Test for Heteroscedasticity and Random Coefficient Variation. Econometrica *47*, 1287–1294.

14. Cribari-Neto, F. (2004). Asymptotic inference under heteroskedasticity of unknown form. Comput. Stat. Data Anal. *45*, 215–233.

15. Cochran, W.G. (1954). The Combination of Estimates from Different Experiments. Biometrics *10*, 101–129.

16. Bulik-Sullivan, B.K., Loh, P.-R., Finucane, H.K., Ripke, S., Yang, J., Schizophrenia Working Group of the Psychiatric Genomics Consortium, Patterson, N., Daly, M.J., Price, A.L., and Neale, B.M. (2015). LD Score regression distinguishes confounding from polygenicity in genome-wide association studies. Nat. Genet. *47*, 291–295.

17. Shin, J., and Lee, S.H. (2021). GxEsum: a novel approach to estimate the phenotypic variance explained by genome-wide GxE interaction based on GWAS summary statistics for biobank-scale data. Genome Biol. *22*, 183.

18. Buniello, A., MacArthur, J.A.L., Cerezo, M., Harris, L.W., Hayhurst, J., Malangone, C., McMahon, A., Morales, J., Mountjoy, E., Sollis, E., et al. (2019). The NHGRI-EBI GWAS Catalog of published genome-wide association studies, targeted arrays and summary statistics 2019. Nucleic Acids Res. *47*, D1005–D1012.

19. Rüeger, S., McDaid, A., and Kutalik, Z. (2018). Evaluation and application of summary statistic imputation to discover new height-associated loci. PLoS Genet. *14*, e1007371.

20. Ge, T., Chen, C.-Y., Ni, Y., Feng, Y.-C.A., and Smoller, J.W. (2019). Polygenic prediction via Bayesian regression and continuous shrinkage priors. Nat. Commun. *10*, 1776.

21. Burren, O.S., Reales, G., Wong, L., Bowes, J., Lee, J.C., Barton, A., Lyons, P.A., Smith, K.G.C., Thomson, W., Kirk, P.D.W., et al. (2020). Genetic feature engineering enables characterisation of shared risk factors in immune-mediated diseases. Genome Med. *12*, 106.
